# Adapting magnetoresistive memory devices for accurate and on-chip-training-free in-memory computing

**DOI:** 10.1126/sciadv.adp3710

**Published:** 2024-09-18

**Authors:** Zhihua Xiao, Vinayak Bharat Naik, Jia Hao Lim, Yaoru Hou, Zhongrui Wang, Qiming Shao

**Affiliations:** ^1^ The Hong Kong University of Science and Technology, Hong Kong, China.; ^2^ AI Chip Center for Emerging Smart Systems, Hong Kong, China.; ^3^ GLOBALFOUNDRIES, Singapore, Singapore.; ^4^ The University of Hong Kong, Hong Kong, China.

## Abstract

Memristors have emerged as promising devices for enabling efficient multiply-accumulate (MAC) operations in crossbar arrays, crucial for analog in-memory computing (AiMC). However, variations in memristors and associated circuits can affect the accuracy of analog computing. Typically, this is mitigated by on-chip training, which is challenging for memristors with limited endurance. We present a hardware-software codesign using magnetic tunnel junction (MTJ)–based AiMC off-chip calibration that achieves software accuracy without costly on-chip training. Hardware-wise, MTJ devices exhibit ultralow cycle-to-cycle variations, as experimentally evaluated over 1 million mass-produced devices. Software-wise, leveraging this, we propose an off-chip training method to adjust deep neural network parameters, achieving accurate AiMC inference. We validate this approach with MAC operations, showing improved transfer curve linearity and reduced errors. By emulating large-scale neural network models, our codesigned MTJ-based AiMC closely matches software baseline accuracy and outperforms existing off-chip training methods, highlighting MTJ’s potential in AI tasks.

## Teaser

Magnetoresistive in-memory computing achieves software-level accuracy through an off-chip adaptive training method.

## INTRODUCTION

Traditional general-purpose digital computers use the Von Neumann architecture (*1*), which features separate memory storage and an arithmetic logic unit and has been highly successful over the past century. However, the growing disparity between the processor speed and memory access time has pushed Von Neumann machines toward a memory bottleneck in recent years (*2*, *3*). In addition, the transistor size is approaching its physical limit, slowing down Moore’s law. Thus, the research committee and industry are actively developing new architectures, devices, and materials to continue the markedly growing trend of computing devices.

Analog in-memory computing (AiMC) with memristors addresses the memory bottleneck by using Ohm’s law and Kirchhoff’s current law to perform efficient multiplication and accumulation (MAC) operations. Memristors are emerging nanoscale tunable resistors that can be achieved using various physical principles. When arranged in crossbar arrays, memristors store neural network parameters as conductance values, enabling highly efficient MAC operations, which are the most intensive operations in deep learning. Memristors’ colocation of memory and processing helps mitigate the massive data shuttling between memory and processing units.

**Fig. 1.**
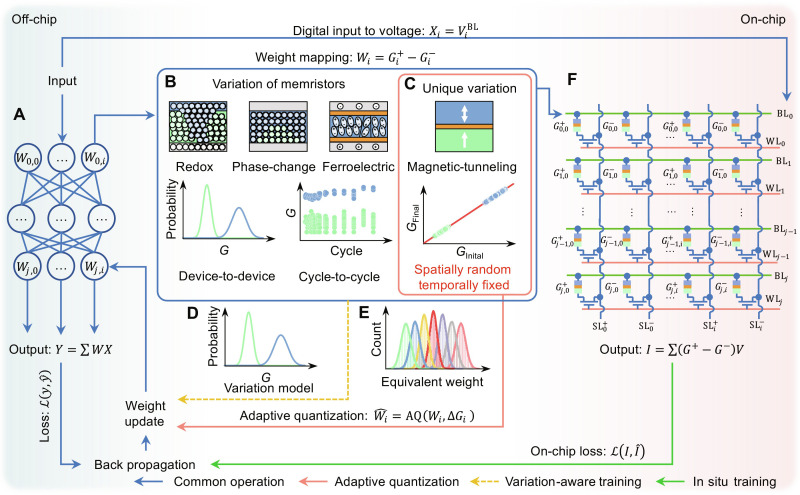
Addressing the design gap in AiMC systems due to memristor device variations. AiMC provides a promising solution for efficient MAC operations. (**A**) A pretrained neural network is compiled to map the weights as conductance in a crossbar array, and doing analog MAC efficiently. However, as in (**B**), memristor variations can lead to inaccurate weight-conductance mappings. (**C**) Magnetic-tunneling memristor features a unique variation, despite of conventional variation-aware training (VAT) based on statistic model as in (**D**). The spatially random yet temporally fixed feature enables (**E**) an off-chip adaptive quantization method to adjust the weights according to the conductance shift of the devices. (**F**) In situ training adjust the weights based on the on-chip loss iteratively. This method, however, requires frequent writing and reading of memristor devices, resulting in reduced device endurance. Although it has the highest accuracy, an off-chip calibration method is still a sought-after goal.

However, stochasticity associated with memristor programming and analog circuit noises can distort the MAC result, leading to inconsistent computational outcomes. As illustrated in Fig. 1, errors arise from imprecise weight-conductance mapping to memristors, such as in Fig. 1B, phase change memory (*4*–*6*), resistive random-access memory (*7*–*9*), ferroelectric field-effect transistor (*10*, *11*), and magnetic random-access memory (MRAM) (*12*–*15*), due to device-to-device (DtD) variations caused by imperfect fabrication processes. Moreover, cycle-to-cycle (CtC) variations in devices and circuits result in discrepancies between cycles, even within the same device (*8*, *9*). Consequently, the performance of deep neural networks (DNNs) may degrade or even become entirely corrupted on memristor-based hardware (*16*, *17*).

Despite the advancements driven by emerging nonvolatile memories, the AiMC is often hindered by the variation in these devices. Thus, reducing the device variation efficiently by the device itself is an important milestone in the hardware (*5*, *6*). Nonetheless, addressing this challenge requires more than a singular focus on software or hardware improvements. The disconnect between software and hardware often results in limited gains from these naïve approaches (*18*–*20*). Thus, codesign schemes have emerged as crucial strategies for bridging the gap between algorithms and devices. Researchers have developed statistical and systematic models to abstract discrepancies in the crossbar array (Fig. 1D), training DNNs to be robust against these discrepancies via variation-aware training (VAT) (*17*, *21*). However, these models are often insufficient in predicting the random conductance variation of memristors, leading to only modest accuracy improvement. An alternative approach, in situ training (*22*, *23*), directly calculates errors on-chip, incorporating discrepancies into the backpropagation process and updating DNN model weights (Fig. 1F). Although in situ training achieves optimal accuracy, the on-chip update cycles increase energy consumption and reduce memristor programming life span.

Concentrating on MRAM-based AiMC, previous studies have explored several methods to enhance AiMC performance. Some work has focused on training DNNs with Gaussian noise to improve model robustness against variations (*24*). Others have examined hardware solutions, such as adequate weight mapping methods (*25*) and readout circuits (*20*, *26*, *27*) that can reduce systematic offsets. Codesign schemes that model the statistical variation behavior of the AiMC chip based on measurements and train the DNN models can result in more accurate computation outcomes compared to approaches focusing solely on software or hardware (*28*). To achieve optimal performance, in situ learning methods that include the hardware in the training process are used (*29*), albeit at the cost of increased energy consumption and reduced device endurance. While these methods demonstrate the promising performance of MRAM AiMC, achieving accurate MRAM AiMC in experiments without on-chip training cycles remains a challenge (*30*).

Here, we first present a hardware-software codesign for the unique spatially random yet temporally fixed device variation of magnetic tunnel junction (MTJ) using mass-produced foundry-fabricated spin-transfer torque MRAM (STT-MRAM) featuring ultralow variation. The randomly distributed and stable switching DtD variation (Fig. 1C) of STT-MRAM inspires us to develop an adaptive quantization AiMC scheme (Fig.1E). We perform array-level quantitative characterization of the transfer function in MRAM-based AiMC systems with and without adaptive quantization. The adaptive quantization method takes advantage of the spatially random yet temporally fixed variation of STT-MRAM devices, requiring minimal additional ex situ training to achieve highly accurate inference comparable to in situ training methods. By emulating the adaptive quantization on a larger scale and with MRAM-based AiMC of varying device qualities, we demonstrate the broad applicability of this method for AiMC systems fabricated with MTJs, regardless of the stochasticity strength of device variations and the scale of artificial intelligence (AI) tasks.

## RESULTS

### Characterization of STT-MRAM

**Fig. 2.**
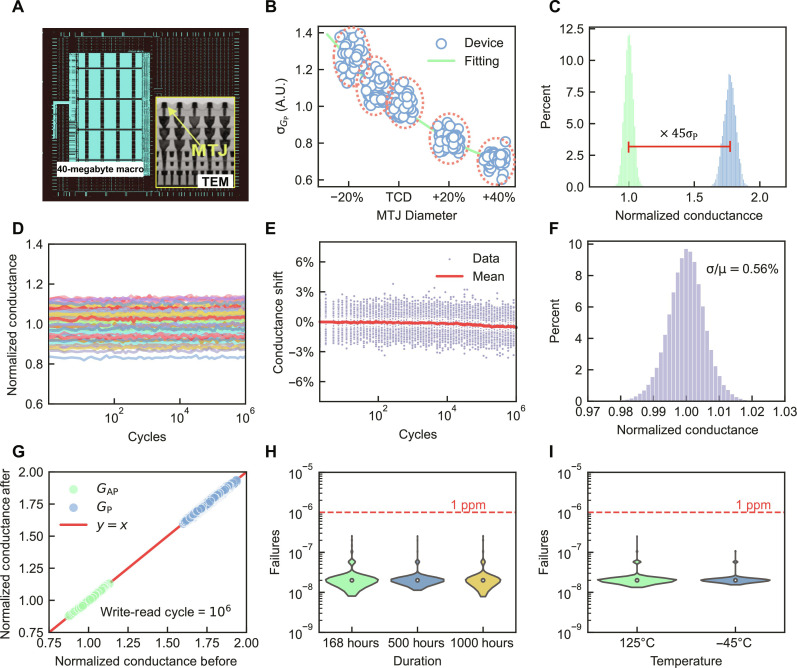
Device characteristics of STT-MRAM. (**A**) 40-megabyte 22FDX STT-MRAM layout. The inset shows the cross-sectional TEM of MTJ arrays embedded between two metal layers. (**B**) The DtD variation increases with the scaling down of the MTJ diameters. We characterized the following device variation using the devices fabricated with target critical dimensions (TCDs). (**C**) MTJ conductance distributions of parallel (P) and antiparallel (AP) states from 128-kilobyte sub-arrays showing the DtD variation of the MRAM array. (**D**) The subarray that continuously repeats reads and writes for 1 × 10^6^ cycles. The conductance is measured after every intermediate cycle. (**E**) The conductance shift for each MTJ in the subarray compared with its initial value during the cycle test, the average conductance shift at 1 million cycle is below 0.6%, and (**F**) The CtC variation statistic of the subarray in the cycle test, the CtC variation is significantly smaller than the DtD variation, which is an essential variation feature for adaptive quantization. (**G**) The conductance before and after the cycle test of two states, all the devices’ conductance values are unchanged, and no device failure was observed during the cycle test, indicating a good endurance and high stability of the MRAM device. (**H**) Wafer median retention bit flip rate tested for 1000 hours; the flip rate is well below 1 ppm. (**I**) Read disturbance rate tested at 125°C and −40°C for 2.8 × 10^6^ read cycles.

We fabricated STT-MRAM using a foundry-compatible 400°C back-end-of-line 22FDX platform to assess variations within MTJ arrays (Fig. 2A). The extent of these variations observed in MTJ arrays fabricated under identical technology nodes is highly related to the size of the MTJ. As illustrated in Fig. 2B, we fabricated five batches of MTJ devices, each with different diameters. The variation in these batches increased with the decreasing device diameter. Therefore, smaller MTJ sizes are favored for larger integration at the cost of increased variation. This finding presents an important challenge to AiMC design schemes aiming to mitigate the increasing discrepancy associated with higher MTJ density.

In the AiMC scheme, the weights are represented by the conductance of the cells in the crossbar array. Thus, the properties of the MTJs are preferably analyzed in terms of their conductivity. We measured the tunnel magnetoresistance (TMR) of the fabricated MTJs. Because of inevitable thin-film inhomogeneity during the fabrication process, the conductance of parallel/antiparallel (P/AP) states forms distributions around ideal values (Fig. 2C). We further subjected the fabricated MTJs to 1 million write and read cycles (Materials and Methods) to continuously change the MTJ state. By recording the conductance of the MTJs for intermediate cycles, we quantified the conductance change across their entire life span. The DtD variation was observed in the recorded conductance of the MTJs (Fig. 2D). The CtC variation is highlighted by isolating and presenting only the conductance shift of each MTJ (Fig. 2E). Statistical analysis of the CtC variation (Fig. 2F) reveals that it is considerably smaller than the DtD variation, suggesting that the latter dominates the overall variation within the MTJ array. Compared with the same or different emerging nonvolatile memories, our devices have ultralow variations without calibration (table S1), which avoids the negative impact on computing efficiency.

Notably, the MTJs demonstrated robust endurance over 1 × 10^6^ write-read cycles, with the conductance before and after the test remaining unchanged (Fig. 2G). This indicates that the factors influencing TMR remain relatively stable after fabrication. Once the devices are fabricated, the DtD variation is spatially random yet temporally fixed. This insight allows us to reduce overall variation by eliminating the temporally fixed DtD variation through properly designed hardware circuits and algorithms. This enables the deep learning model to focus on managing the less substantial, truly random CtC variations.

We also tested data retention and read disturbance under various read temperatures (Materials and Methods). The bit error rate remained comfortably below 1 part per million (ppm) for all devices (Fig. 2H) after the 1000-hour retention test at the worst-case scenario temperature of 150°C, emphasizing the excellent data retention capability of the MTJs. The read disturbance rate under test temperatures of 125° and −40°C remained well below 1 ppm (Fig. 2I). These results confirm that the MTJ is sufficiently reliable for storing off-chip optimized DNN parameters and can be securely accessed multiple times for inference purposes.

### Enhancing the accuracy of AiMC MAC operations via adaptive quantization

**Fig. 3.**
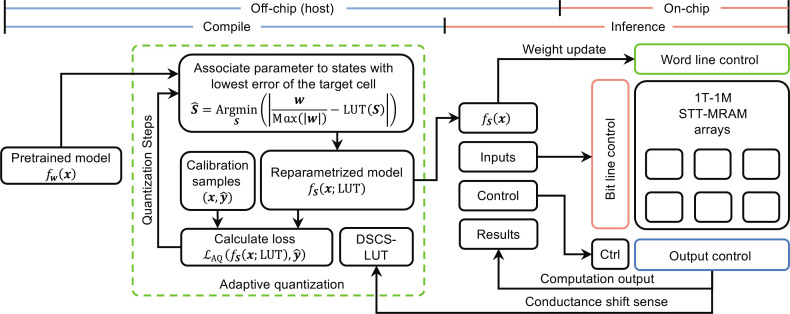
Adaptive quantization system and flow. A pretrained model is compiled with adaptive quantization and the weight is associated with the states of the devices for inference. The external host controls the macro, and the on-chip sensing circuits are bypassed. External high-precision measurement tools obtain the output from the subarray for detailed analysis.

Our measurement scheme uses an off-chip system (Host) to apply control and measurement signals to the chip. As illustrated in Fig. 3, an adaptive quantization process is executed in the compile stage the first time deploying a model. First, a conductance shift sensing process will capture the conductance shift of each cell in the crossbar array and store the results in a device-specific conductance shift lookup table (DSCS-LUT). Second, the parameters of the pretrained model *f*_***W***_(***x***) will be associated with states of the target cell in the chip by picking up the states with the minimum difference between the parameter value and the cell value, denoted as *f*_***S***_(***x***). Then, a few calibration samples from the training data are used to calculate the loss of reparametrized model and optimize the parameters. This process will iterate for several steps to improve the performance of the model. Last, the parameters of the model are stored as the state of the cells for inference.

**Fig. 4.**
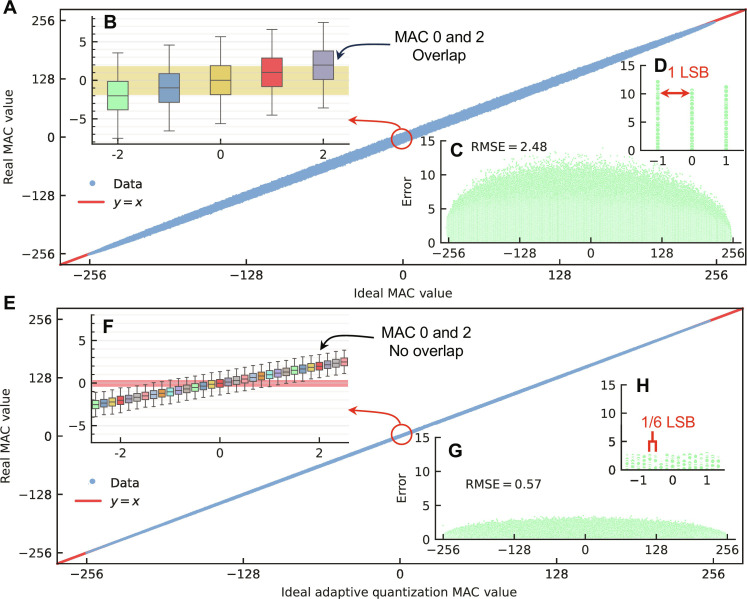
MAC operation transfer curve. (**A**) The transfer function of conventional quantization, where the real MAC value is converted from the output currents. The top left inset (**B**) shows that overlap happens between adjacent MAC values. The bottom right inset (**C**) shows the RMSE of each MAC value, whereas the zoom-in plot (**D**) shows the minimum resolution is 1 LSB. (**E**) The transfer function of adaptive quantization shows an improved linearity. The top left inset (**F**) shows that the overlap between MAC values is shrunk to a more nearby region. The error plot in the bottom right inset (**G**) supports that adaptive quantization can reduce the MAC error to 0.57 RMSE. The minimum resolution in the zoom-in plot (**H**) is improved to 1/6 LSB for a 3-bit conductance shift sensing-based adaptive quantization.

To evaluate the effectiveness of adaptive quantization, we measured the transfer curve of basic MAC operations with and without adaptive quantization. The variation of the transfer curve depends on the input and weights (note S5). For clear visualization, we fixed the inputs to be all ones and randomly tested 1 × 10^5^ combinations of weights to get the transfer curve in this experiment. Figure 4A displays the MAC test results for a conventional AiMC. In this case, we directly measure the output current of a differential pair to obtain the transfer curve and then calculate the real MAC value based on this measurement. Ideally, the real MAC value should range from −256 to 256, represented solely by integers. However, because of discrepancies in the crossbar array, the output current exhibits inaccuracy, resulting in a distribution for each integer MAC value. Figure 4B plots the detail in the range of −2 to 2, which shows overlaps between MAC values and their adjacent values, potentially leading to error detection within the readout circuits. In Fig. 4C, the average root mean square error (RMSE) of the conventional MAC is 2.48 lowest significant bit (LSB), indicating a high probability of each MAC operation’s output being confused with 2 to 3 nearby values. This faulty MAC operation potentially reduces the accuracy of the DNN models executed in the AiMC systems.

Figure 4E presents the transfer curve of the MAC operation with adaptive quantization. In this experiment, the conductance shift value of the devices within the array is down-sampled to mimic the behavior of a 3-bit conductance shift sensing analog-to-digital converter (ADC). This makes the smallest resolution of the MAC reduced to 1/(2^*n*_ADC_^ − 2) = 1/6 LSB (note S4a). The same hardware using adaptive quantization yields a far more linear transfer curve than the conventional approach. Figure 4F plots the detail of the same area in Fig. 4B, revealing that the adaptively quantized MAC values exhibit reduced nonlinearity. With adaptive quantization, the output variation for a MAC value of 0 significantly reduces, resulting in no overlap with the ideal MAC value of 2. Within the 25 to 75% confidence interval, the adaptively quantized MAC operation does not overlap with a nearby 1 LSB value, thus reducing the likelihood of fault detection. In addition, the insertion error plot in Fig. 4G indicates that the RMSE of adaptive quantization is merely a fifth of that of the conventional MAC. A further detailed plot (Fig. 4H) shows that adaptive quantization can yield a smaller resolution of the MAC operation compared with the conventional scheme shown in Fig. 4D. Our method is effective because the CtC variation of MTJs is significantly smaller than the DtD variation (notes S6 and S7).

The cost of implementing such an adaptive quantization scheme lies in three aspects: the cost of conductance shift sensing, the storage of the DSCS-LUT, and the additional quantization steps. We evaluated how the accuracy improvement of the adaptive quantization method is related to the implementation cost (note S4) and found that the increase in accuracy is substantial when sensing precision is 3 bits, and the improvement of higher precision ADC is marginal. Thus, there is no extra cost in implementing a chip area for high precision ADC and memory to store a large LUT. This indicates a low overhead for implementing our method.

### Adaptive quantization in real-world AiMC tasks for DNN models

**Fig. 5.**
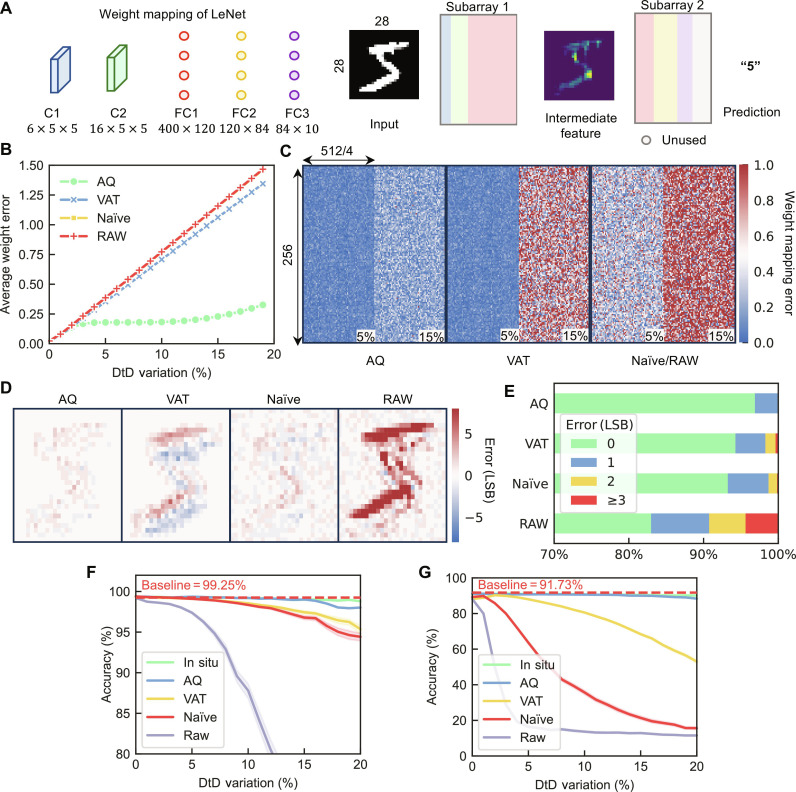
Codesign schemes in real-world AI applications. (**A**) To map the 2-bit weights in the 256 × 512 array, the maximum capacity of the array is 32,768. The parameters are mapped into two arrays and stored for inference tasks. The error is defined as the difference between measured conductance and expected conductance. In the following tests, the CtC variation is fixed at 0.5% and the DtD variation is varying. (**B**) The average weight mapping error of adaptive quantization and other methods, and the weight mapping error of all the methods in the first subarray is plotted in (**C**). (**D**) The output feature error map of the first convolutional layer and (**E**) the error distribution represented in LSB shows that codesign schemes can reduce the computational error, the adaptive quantization method is especially efficient in reducing significant outliers in the output at 5% DtD variation. The overall performance in the (**F**) MNIST tasks shows that most codesign schemes can maintain high accuracy when the variation of the devices is low. A substantial accuracy drop appears when the variation is high. However, in (**G**) the performance of the models is easily affected by the device variation. Only adaptive quantization can achieve in situ training level accuracy.

To analyze the effectiveness of the proposed adaptive quantization in facing the challenge of increased DtD variation in shrunk devices for higher integration density and the varying complexity of real-world applications. We used a detailed circuit simulation to project the performance of our proposed adaptive quantization scheme for them (Materials and Methods). The simulations used device models calibrated with fabricated MRAM macros. We aimed to demonstrate the effectiveness of the adaptive quantization method and therefore included the naïve, VAT, and in situ training methods in the emulation. We first used LeNet on the Modified National Institute of Standards and Technology (MNIST) (*31*) dataset, which features two convolutional layers (150 and 2400 parameters) and three fully connected layers (105,000 parameters in total). These parameters are stored in the MRAM macro for inference (Fig. 5A).

One of the primary challenges in deploying a DNN model to the AiMC system involves accurately storing the parameters in the crossbar array. Device discrepancies can cause the conductance to deviate from the ideal value, even when the MTJ is programmed to the targeted state. Therefore, we gathered the average weight mapping error using different schemes to evaluate how these methods could mitigate the weight mapping error. As shown in Fig. 5B, the adaptive quantization method can adjust the weight according to the sensed conductance shift of the targeted cell, thereby effectively reducing the weight mapping error. When the device variation exceeds the threshold of the conductance shift sensing circuits, the model becomes aware of the device’s conductance shift. The error remains low until the device variation surpasses the upper bound of the sensing circuits and remains significantly lower than other methods (Fig. 5C). Other methods, like the raw mapping method, map the weights directly as the conductance without optimization, leading to a linear increase in weight mapping error as device variation increases. The naïve approach shows a similar error increase, as it primarily enhances the model’s performance by increasing its weight sparsity, thus making it more robust to weight perturbation. The codesign scheme, VAT, train the model using hardware abstractions, reducing the weight mapping error slightly. The statistical model and hardware abstractions do not provide sufficient information in the training process to prevent errors while transferring parameters to the hardware representation. Consequently, the weight error still increases linearly with variation.

We recorded the output from each channel in the first convolution layer and plotted the error between the expected and actual output with a 5% DtD variation for each scheme (Fig. 5D). The output error arises from several factors, including the weight mapping error caused by device variation and noise in the circuits. The adaptive quantization method delivered the lowest error rate among all the methods. As shown in Fig. 5E, the average absolute error value (defined in note S6) of the naïve scheme is 0.71, with approximately 82% of the outputs being correct. VAT reduces the error to 0.51 and increases the correct outputs to 93%. The adaptive quantized model further reduces the error value to 0.23 and correct outputs to 96%. The adaptive quantization scheme also reduces significant outliers in the output and minimizes the SD of the error distribution, preventing the accumulation of extreme error values across layers, which could compromise the model’s inference.

The overall performance is plotted in Fig. 5F. A 2-bit weight and 2-bit activation model trained with a conventional quantization method can achieve a performance of 99.25% accuracy at zero variation. In the presence of variation, the adaptive quantization method can deliver comparable accuracy to in situ training, with an accuracy drop of less than 1% under device variation as large as 20%. Other off-chip optimization methods maintain a low accuracy drop while the device variation is small, but the accuracy drop increases exponentially as the variation increases. For instance, under 20% device variation, VAT can only achieve 95.35% accuracy (baseline −3.9%). The drop is more than 5× higher than adaptive quantization. Extending this comparison to codesign schemes based on different technologies and calibration methods (table S2), our method can achieve optimal accuracy without on-chip calibration cycles, which can greatly reduce the overhead of the calibration. The performance of our method can even outperform the model without variations due to the enlarged model capacity (note S3) in some scenarios.

The wide range of AI tasks demands the development of models with diverse structures and complexities. The accuracy of these AI tasks is sensitive to the model structure and parameter redundancy. We deployed our method using the larger network-in-network (NIN) (*32*) model on the more complex CiFar-10 (*33*) dataset (Materials and Methods). As shown in Fig. 5G, the accuracy of each method drops more significantly in the CiFar dataset due to reduced model redundancy in more complex tasks. The adaptive quantization method still achieves in situ training comparable performance of 88.38% (baseline −3.45%) under the highest device variation. For other methods, the accuracy drop is substantial even under small device variations and increases with increasing device variation (VAT can achieve 52.99% under 20% device variation). This supports the adaptive quantization method’s ability to address the challenges of deploying AiMC systems on larger AI systems. First, adaptive quantization does not solely rely on the robustness introduced by model redundancy, which makes it outperform other methods when the AI task is more complex. Second, it is not sensitive to changes in DtD variation, allowing it to be deployed on AiMC systems fabricated with high integration density devices.

## DISCUSSION

In this study, we present an adaptive AiMC scheme that dynamically quantizes parameters during off-chip training. This approach capitalizes on the device variation characteristics observed in multiple foundry-fabricated MRAM macros. The proposed scheme notably enhances the linearity of the MAC transfer curve in the experiment, providing substantial accuracy improvements to the AiMC. A scaled-up emulation further validates the effectiveness of the adaptive quantization method. Implementation of the proposed scheme necessitates only a conductance shift sensing circuit for hardware support, which eases the detection of device conductance shifts. With minimal software and hardware overheads, DNN models optimized using adaptive quantization can achieve highly accurate inferences on MRAM-based AiMC systems.

The adaptive quantization method, devised based on distinctive MRAM variation characteristics, fully exploits these features. This strategy combines design principles from dynamic range quantization schemes and uses inherent hardware properties to enable highly efficient computation. In addition, it maintains high accuracy when applied to memory devices of diverse densities and models with varying complexities. By leveraging intrinsic hardware variation and promoting codesign between software and hardware, this method offers potential for application to other use cases and memory devices, with suitable adaptations.

## MATERIALS AND METHODS

### MRAM array structure and fabrication

The MTJ stack was deposited using magnetron sputtering on 300-mm Si wafers. The free layer (FL) and reference layer (RL) consist of CoFeB-based ferromagnets, with the RL pinned by synthetic antiferromagnet layers. The studied MTJ devices were cylindrical and featured five nominal target critical dimensions (TCD): TCD-20%, TCD-10%, TCD, TCD + 20%, and TCD + 40%. The maximum MTJ diameter is <100 nm. Positive polarity is defined by the current flow from RL to FL. MTJ devices are set to the P and AP states by positive and negative voltages, respectively. The fabricated memristor array adopts a 1-transistor–1-MTJ structure, with all transistors, metal connections, and vias fabricated using a standard 22FDX complementary metal-oxide semiconductor (CMOS) foundry technology node. The MTJ is deposited on top of the CMOS devices as a back-end of the device. The 40-megabyte MRAM macro is composed of multiple 5-megabyte sub-banks. To write to a memory cell, a high control voltage is applied to the word line (WL) to activate the access transistor to the STT-MRAM cell, while a write voltage is applied to the bit line (BL) and source line (SL) to generate a write current for switching the MTJ state. After the write period, data are programmed into the memory cell (P for 1 and AP for 0), with data-programmable parallel in the row direction for write operations. To read a memory cell, a high control voltage is applied to the WL to activate the access transistor, and a read voltage is applied to the BL, generating a read current on the SL.

### STT-MRAM variation characterization

To measure the MTJ conductance characteristics in the array, crossbar arrays are connected to external circuits for writing and measurement. A write pulse with a 200 ns duration is applied through the BL driver to the target MTJ, with the write current direction determining the MTJ resistance state. After each write pulse, a read pulse of 200 ns with ~0.1 V is applied to measure the resistance of the targeted device. In the cycling test, each write cycle consists of two programming pulses (negative pulse and positive pulse), and the bit conductance is measured after a certain number of programming pulses. The arrays are tested for 1 million cycles with solder reflows compatible MTJ stack at −40°C and ≥1 × 10^6^ cycles without solder reflows compatible stack for extending the cycling endurance. A resistance bitmap is obtained by setting the entire array to the low resistance state (LRS) and applying read pulses to the array, with 32 by 32 subregions randomly selected within the subarray (fig. S8). The resistance is normalized using the mean resistance of all measured devices at LRS. In reliability testing, the STT-MRAM packages are tested as per standard JEDEC reliability conditions. To test data retention, 77 units × 3 lots are programmed to P and AP states (half of the memory of the macro for each state) and kept at the worst temperature corner (150°C) data retention fails have been monitored at 168, 500, and 1000 hours. The read disturb rate is estimated from the high (125°C) and low (−40°C) temperature operating life tests with continuous reading up to 2.4 million cycles for 1000 hours. The measurement data of Fig. 2 are tested on devices with TCD.

### Weight mapping in the crossbar array

Unlike other analog nonvolatile memories, which can map arbitrary precision parameters into multiple analog devices via analog bit-slicing (*34*), MRAM is predominantly utilized as a binary device (*19*, *28*, *35*, *36*). Consequently, a ternary-encoded multiple-bit weight mapping method based on the ternary differential bit-cell (1, P-AP; 0, AP-AP; and −1, AP-P) is used (note S1). As illustrated in fig. S9, an *n*-bit weight is stored in adjacent n columns in the same row. The real value of this weight *W* is calculated using Eq. 1

$$W=\sum_{i=1}^{n}G_{i}\times 2^{i-1}\;\;G\in \left(-1,0,1\right)$$ (1)

Here, *G_i_* is the differential conductance of each differential pair, normalized to *G* ∈ (−1,0,1) by the standard conductance value of each state to represent the signed integer value in Eq. 1. Unlike other ternary bit weight mapping methods that use two’s complement number representation, we use an encoding method that is a variant of binary coded decimal (BCD) code as the number representation scheme to enable our adaptive quantization algorithm on hardware. In our AiMC architecture, each differential cell (bit) has three states with signed values. Accordingly, a signed BCD encoding method is employed. This encoding method allows only the conductance state *G* of each bit for a single weight to have the same sign to avoid an equivalent representation of the same value. For example, if *W* = 1 and *n* = 2, the conductance state of the two differential pairs can be either (*G*_0_, *G*_+_) or (*G*_+_, *G*_−_) since both combinations result in *W* = 1 in Eq. 1. Thus, the total state number of an *n*-bit parameter is 2^*n*+1^ − 1.

### AiMC operation

For AiMC, multiple rows on the same column can be accessed simultaneously to perform analog MAC operations (*37*). To reduce the control complexity needed to generate accurate multiple input voltage levels, the inefficient layout area, and extra power consumption caused by a high-precision input digital-to-analog converter (*38*), only binary values are applied to the BL as inputs. In this AiMC scheme, multiple-bit calculations are performed in multiple cycles (fig. S9). Each bit’s temporal result is converted to digital values by the readout ADC and stored in the shift registers. The complete results are obtained by adding the temporal results bit by bit. As described by Eq. 2 and in fig. S9, the *n_a_*-bit inputs ***IN*** and *n_w_*-bit weights ***W*** require *n_a_* cycles to complete the MAC operation, during which the ADC converts the analog temporal results to digital values [Quant_ADC_(·)] after a single MAC operation of one input bit

$$Y=\sum_{b=1}^{n_{a}}\sum_{a=1}^{n_{w}}\text{Quan}t_{\text{ADC}}\left(\sum_{i=1}^{m}W^{i}\left[a\right]\text{IN}\left[b\right]\right)2^{a+b-2}$$ (2)

### Variation in magnetic-tunneling memristor crossbar array

The conductance variation caused by multiple factors in design, fabrication, and operation can be divided into two categories: DtD variation (∆*G*_D_) and CtC variation (∆*G*_C_). The overall conductance variation ∆*G* can be considered as the combined contribution of both variations. Although both ∆*G*_D_ and ∆*G*_C_ exhibit stochastic properties, only ∆*G*_C_ is a true stochastic noise, demonstrating its randomness both spatially and temporally. ∆*G*_D_ only exhibits its stochasticity spatially. Thus, the ∆*G*_D_ leads to a static conductance shift on each device, so the total variation for a single device ∆*G* can be written as

$$∆G=\left\{ \begin{array}{ll} ∆G_{C}\sim N\left(\mu_{D|P},\sigma_{C|P}^{2}\right) & P\;\text{state}\\ ∆G_{C}\sim N\left(\mu_{D|\text{AP}},\;\sigma_{C|\text{AP}}^{2}\right) & \text{AP}\;\text{state} \end{array}\right.$$ (3)

Here, μ_D_ is shifted by the conductance *G*_AP_ and *G*_P_ for this device after considering ∆*G*_D_ as a known static shift in the conductance value. The stochasticity of device variation is primarily determined by the ∆*G*_C_, following the normal distribution. Considering the conductance shift caused by DtD variation of each device on the chip allows the software to use statistical models to overcome the influence of ∆*G* with relatively smaller stochasticity ($\sigma_{C}^{2}$), which is essential for the multiple-bit weight scheme in AiMC. As described by Eq. 2, the different bits of the inputs and weights do not equally contribute to the final MAC results. Assuming the weights are affected by a noise with the power of σ^2^, for an MAC operation with *n*-bit input and weight, the weight on the *i*th bit contributes 2^*i*−1^σ^2^ variation to the partial MAC result. The partial MAC result is then shifted and added to the final MAC result. The total variation in the final MAC result suffers from a noise with the power of (2^2*n*^ − 1)σ^2^/3. This indicates that the noise in the multiple-bit AiMC system accumulates exponentially with the bit number, making it essential to control the overall variation at a lower level.

### Conductance shift sensing

To determine the conductance shift for each device, a sensing method is employed to obtain the μ_D_ in Eq. 3. The differential pairs can be accessed individually by activating a single row. Subsequently, the built-in self-test circuits are configured to sensing mode, using corresponding reference voltages during the sensing process. The entire array is set to *G*_−_, *G*_0_, and *G*_+_ to measure the conductance shift of each state. The DtD variation results in a conductance shift distribution centered around the standard conductance *G*_STD_. A properly biased conductance sensing circuit can detect this distribution, and the digital readout values represent a quantized distribution of the conductance shift. This allows for determining each cell’s shifted conductance under the targeted state. Following conductance shift sensing, the host records the DSCS-LUT for each cell, enabling the adaptive quantization algorithm to account for variations during the requantization. Table S3 provides an example of a DSCS-LUT for a differential cell sensed by high-precision external measurement equipment and down-sampled as a 3-bit ADC.

### Weight quantization

In conventional quantization methods (*39*), the arbitrary bit quantization of the proposed ternary encoded weights can be expressed as

$$Q=\frac{1}{2^{n}-1}\times \text{Round}\left(\left(2^{n}-1\right)\left\{\frac{\text{tanh}\left(x\right)}{\text{Max}\left[\left|\text{tanh}\left(x\right)\right|\right]}+1\right\}\right)-1$$ (4)

In Eq. 4, ***x*** represents the floating number weight, while ***Q*** denotes the *n*-bit quantized weight. ***Q*** naturally falls within the range [−1,1] with 2^*n*+1^ − 1 fixed values. The Round(·) function takes the nearest integer around its input. The quantization method for activations and weights follows Eq. 4, except that activations use a regular binary encoding method instead of the signed BCD encoding for the weights.

The conventional quantization method functions well in the digital domain. However, in analog computing, device variations prevent weights represented by device conductance from being evenly distributed within the given range even without random CtC analog noise. Therefore, adaptive quantization is introduced to bridge the gap between conventional quantization and nonuniform device conductance (note S3). Similar to the traditional approach, the weight is first calculated to determine its location within a specific region

$$\hat{S^{i}}=\underset{S^{i}}{\text{Argmin}}\left[\left|\frac{w}{\text{Max}\left(\left|w\right|\right)}-\text{LUT}\left(S^{i}\right)\right|\right]$$ (5)

$$G=\text{LUT}\left(\hat{S^{i}}\right)$$ (6)

In Eq. 5, ***S*** represents the state number of the quantized weight. The weights are calculated using Eq. 5 to determine the closet state section (***S****^i^*) in which ***w*** falls. The real value of the quantized weight is then obtained by checking the equivalent value of the conductance of the corresponding device in the DSCS-LUT as in Eq. 6. Although this quantization function is discontinuous, the straight-through estimator (*40*) method remains compatible with the backpropagation of the adaptive quantization process. When the model is deployed for inference, the parameters only record the device state of the corresponding device, and the device initiation process is naturally equivalent to the lookup table matching. Consequently, the only additional data access cost for the adaptive quantization method is the lookup table checking during training.

### Magnetic-tunneling memristor AiMC emulator

Assessing the detailed device variation characteristics in a fully integrated CMOS AiMC system is challenging. As a strategic response to this challenge, we fabricated several STT-MRAM arrays, as previously detailed. This gave us unrestricted access to all devices in these arrays to facilitate various reliability tests. We then incorporated the measured behavior of the devices from the fabricated arrays into an emulator which is designed to simulate AI tasks within AiMC systems. This emulator uses an experimentally calibrated STT-MRAM device model to simulate the device behavior and match the characteristics of the emulator’s array with the fabricated arrays. In the emulator, we configured the arrays into an in-memory computing macro by pairing MTJs in two adjacent columns in the measured arrays to form a single differential cell. This process yielded a total of 256 by 256 (65,536) differential cells in a single array. The emulator allowed us to scrutinize a wide range of factors that could potentially influence the performance of the AiMC scheme (note S2, S5, and S6). The factors we examined included the critical dimension of the MTJ, array size, output circuit design, etc. It offered profound insights into the detailed mechanisms of the proposed scheme. In addition, it supported multiple operation kernels for DNN inference, including the convolutional layer and the fully connected layer. This approach has provided us a more insightful analysis of how each device’s variation could potentially affect the system’s overall performance. To evaluate the AiMC emulator, we used the same measurement method as the MAC transfer curve test and experimentally tested the MNIST task using LeNet-5 on the TCD devices. A comparison between the emulator’s and the experiment’s results is plotted in fig. S10, indicating that the emulator can successfully emulate the hardware behavior.

### Image classification task setup

We used the LeNet-5 architecture, a representative convolutional neural network (CNN), to classify handwritten digits from the MNIST dataset. The MNIST dataset is a benchmark dataset featuring 60,000 training samples and 10,000 testing samples of grayscale images with dimensions of 28 × 28 pixels. Each image represents a single handwritten digit ranging from 0 to 9. For models with more parameters, we used the NIN and deployed it on the CiFar-10 dataset. The CiFar-10 dataset is an image classification dataset with 10 image categories. The input images are color images sized 32 × 32 and separated into 50,000 and 10,000 images for training and testing, respectively. Before feeding the datasets into the models, we preprocessed the data by normalizing the pixel values to the range [0,1] and zero-centering the dataset by subtracting the mean pixel value. In addition, we resized the MNIST images to 32 × 32 pixels to match the LeNet-5 input size. Optimization on both tasks was performed using the AdamW (*41*) optimizer with a 1 × 10^−3^ initial learning rate that decreased at cosine annealing decay for 100/500 epochs, respectively. Both models are quantized to 2-bit weight and activation for deployment.

Since the AiMC scheme aims to accelerate MAC operations, only the convolutional layer and fully connected layer are processed in the AiMC macro. Meanwhile, other types of layers, such as the pooling layer and batch normalization layer, are processed in the host. To map a layer to the AiMC macro, if the input channel number multiplied by the kernel size is smaller than the row number of the array (256), this kernel will be directly processed in the IMC macro at once. If the layer is too large to be processed at one time, then this layer will be tiled into smaller pieces to fit the crossbar array size. The intermediate value will be added together by the host when all the partial results are obtained, as shown in fig. S11A.

### Training process

Existing training methods that explore hardware variation mainly consist of five approaches: (i) adding random noise based on hardware variation statistics (*24*, *42*), (ii) creating a model of the hardware to reduce systematic variation (*17*, *43*, *44*), (iii) modifying the network structure to increase model robustness (*24*), (iv) using redundant devices to reduce random noise in the array (*45*), and (v) directly calibrate or train the model in situ (*46*). In our experiment, we sought to keep the cost of increasing inference accuracy limited to the training phase. Thus, we kept the network structure and size unchanged and did not use multiple-bit cells as redundant copies of a single-bit. The naïve approach trains the model with Gaussian noise during the forward pass. In addition, as suggested by other research, training the model using larger variations than the real value helps the model become more robust. Thus, we gradually increased the gain of the stochastic function from 0 to 2 during the training cycles. For the VAT method, we first measured the transfer function as described earlier and then modeled the stochastic function that approximates the SD of the variation in the transfer function. During training, random noise following a Gaussian distribution and the stochastic function was added to the result each time the AiMC macro was accessed. For the loss function in VAT method, in addition to the regular cross-entropy loss, we also added contrastive loss as an additional term to make the output of the model consistent between different cycles. The contrastive loss between two samples of the models helps the model find a more robust representation of the feature space against hardware noise (*17*, *47*). The trained models were used as the VAT model and the pretraining model of adaptive quantization methods. Since the adaptive quantization process is unique for each AiMC macro, the computation of the model is mortal. Unlike conventional immortal computing schemes where the computation remains the same across different hardware (*48*), the fine-tuning adaptive quantization process is optimized to minimize cost. On the basis of the DSCS-LUT, the parameters of the pretrained models are requantized and fine-tuned to match the conductance of the target device. At this stage, the model has already converged at a local minimum that provides good noise tolerance with decent accuracy. Therefore, only a few epochs (or one shot) are needed to requantize and optimize the model for specific devices (*30*).

## Supplementary Materials

### This PDF file includes:

Supplementary Text Figs. S1 to S11 Tables S1 to S3 References
