# Supplementary material for: Adapting magnetoresistive memory devices for accurate and on-chip-training-free in-memory computing
Source: Sci Adv. 2024 Sep 18;10(38):eadp3710. doi: 10.1126/sciadv.adp3710 (PMC11409953; doi:10.1126/sciadv.adp3710)
Supplement: Supplementary file 1 — Supplementary Text Figs. S1 to S11 Tables S1 to S3 References [file sciadv.adp3710_sm.pdf]

Science Advances
AAAS

Supplementary Materials for

Adapting magnetoresistive memory devices for accurate and on-chip-training-free in-memory computing

Zhihua Xiao *et al.*

Corresponding author: Qiming Shao, eeqshao@ust.hk

Sci. Adv. **10**, eadp3710 (2024)
DOI: 10.1126/sciadv.adp3710

This PDF file includes:

Supplementary Text
Figs. S1 to S11
Tables S1 to S3
References

Supplementary Text

Supplementary Note 1: The Differential Cells

Achieving high efficiency and accuracy in Analog in Memory Computing (AiMC) necessitates quantizing weights and activations into low-bit widths. Ternary bitcells present a compelling option for MRAM-based AiMC, as they can be constructed using two binary memory elements. The balanced ternary representation (-1, 0, +1) is well-suited for certain arithmetic operations, such as addition and subtraction, due to the absence of separate carry or borrow operations. Moreover, the ternary bitcell is more appropriate for multiple-bit scaling (*35*, *49*, *50*). The binary bitcell is unable to represent multiple-bit signed integer values without redundant cells, such as the thermometer code (*20*), which diminishes the information density of the memory devices. In contrast, ternary bitcells can easily be extended to multiple-bit signed values, resulting in higher computational efficiency in AiMC systems.

Another concern in designing bitcells in AiMC systems is the device variations in the memory device. The different conductance shifts due to the device variation and the circuit noise of each cell in the crossbar array reduce the read margin between different states. Differential cells exhibit more resilience to noise and interference than binary cells, as they store information in the difference between two devices rather than a single absolute value. This is particularly beneficial for memory devices with a low on-off ratio, such as MRAM, where the differential cell can double the dynamic range (*51*). This improved robustness can strengthen error resilience for multiply-accumulate (MAC) operations in AiMC systems (*18*).

Supplementary Note 2: IR Drop in the Crossbar Array

The computation of a crossbar array relies on the conductance of the memory devices. Although the wire resistance of the interconnects is typically minuscule at approximately 1 ohm per cell, it can cumulatively have a significant impact on in-memory computing accuracy (*44*, *52*) when the AiMC system is implemented with large arrays (Fig. S1A). The output of the proposed differential cell is obtained by calculating the difference between the positive source line (SL^{+}) and negative source line (SL^{-}). The current on the source line with respect to one cell can ideally be calculated by $I_{SL} = V_{IN}G$. However, due to the presence of wire resistance, the conductance of the target cell is degraded. Consequently, the output current of this differential cell can be expressed as the equation in Fig. S1B.

The wire resistance decreases the conductivity of the cell, subsequently reducing the output yield of the cell, with a strong dependence on the cell position in the array (Fig. S1B). This poses a challenge for increasing the integration scale of AiMC systems since the accuracy of MAC operations and AI tasks can be severely affected by the increased wire resistance in large arrays. Appropriate error detection and compensation mechanisms are necessary to restore data integrity and computation accuracy.

For some memristor devices, the conductance can be tuned with an analog flavor (*22*, *44*), allowing the wire resistance in the array to be modeled and compensated for by adjusting the target conductance of the memristor. However, the MTJ has only two conductance levels, rendering direct adjustments to the device conductance impossible. Instead, the parameters programmed into the device can be adjusted to fit the degraded conductance of the target device. The proposed scheme can adaptively quantize the parameters according to the device-specific conductance shift

look-up table (DSCS-LUT). Each differential cell is accessed and measured during the conductance shift sensing process to obtain the device-to-device (DtD) variation. In the measurement, the sensed conductance shift inherently includes the wire resistance, implying that the DSCS-LUT also contains the necessary information (wire resistance) to compensate for the IR drop through adaptive quantization.

Supplementary Note 3: Model Capacity Improvement Using Adaptive Quantization

Quantized activations and weights in neural networks often limit model performance due to the reduced optimization space compared to floating-point number represented models. In other words, the capacity of quantized models is inherently smaller than that of full-precision models. Recent research has introduced additional parameters to dynamically adjust the step and range of quantized models to address this issue, thereby increasing the optimization space and improving performance (*53–55*). In line with the evolving trend of novel quantization schemes in the computer science community and considering the unique properties of MRAM variation characteristics, we propose an adaptive quantization scheme that leverages the inherent device variations of the MTJs to quantize parameters for efficient and accurate AiMC inference adaptively. Unlike dynamic quantization on digital computers, which requires extra floating-point parameters to adjust the range and step of the parameters, the adaptive quantization scheme can dynamically adjust the parameters using the inherently non-uniform steps and ranges based on the knowledge of the conductance shifts of the AiMC chip's devices. Consequently, no additional floating-point parameter is needed to enlarge the quantized model capacity, reducing the cost of implementing such a scheme in terms of extra storage and computing.

To demonstrate the advantage of utilizing the intrinsic device variation of MTJs in the AiMC, we designed a linear regression experiment with a simple target function, $f(x) = \pi x$. A single-layer perceptron model without a bias term is used in the regression task, as the computation of the synapses can be directly compared to the MAC operation in a single column of the AiMC crossbar array. The difference between the summation of the weights and the target slope, π is the error of the model. The digital quantized model's weight is represented by ternary values (-1, 0, +1), resulting in the best possible regression value of 3 (Fig. S2A). However, when deploying a model with ternary weights to the AiMC macro, the conductance of each cell does not precisely equal the target conductance due to device variation. This variation allows the model to obtain a larger optimization space for better regression. As shown in Fig. S2A, although the weight states are ternary for both digital (-1, 0, 1) and adaptive quantization (P/AP, AP/AP, AP/P), there is a chance for the adaptive quantization in the AiMC to achieve superior results. In this case, an increase in device variation does not necessarily lead to the degradation of the AiMC's performance, as more significant conductance shifts can be combined to achieve better outcomes. We implemented the adaptive quantization scheme on five different device batches (A-E) with varying target MTJ dimensions (CDs) with different device variations (Fig. S2B). The experimental results (Fig. S2C) show that the fitting error increases with the variation if the neuron numbers are small. This is because the optimization space happens to be trapped in a worse condition due to the randomness of the variation. To reduce the chance of the parameter space being trapped, a sufficient number of synapses (devices) are needed. Thus, the regression error decreases when the number of neurons increases. It seems that to ensure the model is not easily trapped in a worse condition, the model should be enlarged to ensure enough parameters are used. The requirement of the parameter number raises another concern about the feasibility of deploying such adaptive methods to smaller

models with fewer parameters. Generally, it is believed that the landscape of the loss functions in finite parameter models is non-convex and has multiple local minima (*56*). This indicates that deep learning models are unlikely to be trapped by the randomly generated parameter exploration space, as shown in the simple linear regression case. The results of the experiment that uses models of different sizes and tasks with varying complexities in the main text demonstrate the generalization of adaptive quantization.

Supplementary Note 4: Adaptive Quantization Implementation Cost

a. Adaptive Quantization Precision

In-memory computing systems employing ternary cells can execute multiple-bit computations by calculating individual bits and combining partial summations through the shift and add operations. The total weight level of the conventional quantization method for a ternary bit cell is given by $2^{n_w+1} - 1$. The proposed adaptive quantization scheme uses conductance shift sensing to measure deviations of the real conductance from the target value. As a result, the output of the Analog-to-Digit Converter (ADC) should be symmetric and centered at the target value, with a total level of $2^{n_{ADC}} - 1$. The conductance shift sensing includes an offset value of a differential cell which can reduce the minimal resolution of the weight to $1/(2^{n_{ADC}} - 2)$ LSB. Adaptive quantization precision is determined jointly by ADC precision and weight bit number. The total level of quantized weights can be expressed by equation (1):

$$W_{level} = 3(2^{n_w} - 1)(2^{n_{ADC}} - 2) + 1 \qquad n_{ADC} > 1 \qquad (1)$$

Here, n_w represents the bit number of weights, and n_{ADC} is the bit precision of the conductance shift sense ADC, which must be greater than 1-bit. The W_{level} rises with the increased precision of weights and conductance shift sensing, as illustrated in Fig. S3A. Increasing n_w necessitates more devices to store additional bits and more cycles to calculate the partial sum. Increasing ADC precision can also increase the number of equivalent weight levels at the expense of a larger conductance shift sensing circuit area. As shown in Fig. S3B, the equivalent weight state number baseline is 1-bit without conductance shift sense (without adaptive quantization), which is 1×. Solely increasing the weight bit number to 5-bit can only raise the state number by 21×. In contrast, increasing the conductance shift sense ADC bit to 5-bit can raise the state number by 60.3×. In our design, we utilized a 2-bit weight with a 3-bit sense ADC, achieving a 36.3× improvement in weight state number compared to the 1-bit baseline.

Recent research has shown that with high precision sensing techniques, a parameter can be mapped into the memristor arrays with arbitrarily high precision for analog computing (*34*). We also evaluated what is the optimal precision for our conductance shift sensing process to balance the tradeoff between accuracy and cost. As illustrated in Fig. S3C, the most significant improvement in weight mapping accuracy occurs when the conductance shift sensing precision increases from 2-bit to 3-bit. Further increasing precision does not yield substantial improvements in weight mapping accuracy. Furthermore, the cost of implementing high-precision sensing circuits escalates exponentially. In this work, we directly reuse the compute ADC as the conductance shift ADC and incorporate additional bias circuits to configure the sensing circuits for different scenarios, achieving negligible extra chip area cost.

b. Cost of Conductance Shift Sensing

As mentioned previously, the extra hardware requirement is negligible for supporting conductance shift sensing. The main cost of this process is the energy consumption and memory required to

store the DSCS-LUT. Suppose the array size is 256×512 (256×256 differential cells). The conductance shift sensing needs to sense the cells under $[-1,0,+1]$ states, this requires 256×256×3=196,608 write and read operations, using 3.4mJ of power (200ns write and read pulse). The storage consumption of the DSCS-LUT using 3-bit ADC is 256×256×3×3=589,824 bit, about 73.7 KB.

Since the adaptive quantization is only executed once while a model is compiled to be deployed to the chip for the first time, the model inference does not rely on the DSCS-LUT. Thus, there are two options for doing the adaptive quantization depending on the restriction of the edge device:

1. Storage bounded:
   The DSCS-LUT is deleted after the adaptive quantization of the model is done. In this case, the conductance shift sensing is needed to obtain a new DSCS-LUT each time a new model is deployed.
2. Energy bounded:
   The DSCS-LUT is stored in the main storage of the edge device. The DSCS-LUT is loaded for use each time a new model is deployed.

Supplementary Note 5: Data Dependency of the Macro

In DNN inference, the combination of inputs and weights is highly random. DtD variation and the intrinsic data dependency of the crossbar array and output circuit design necessitate an exploration of variation distribution in relation to data dependency. Assuming the conductance of a device follows a normal distribution $G \sim N(\mu, \sigma^2)$, the output current of a single column can be expressed as $I = \sum_r x_r G_r$. When the input x of a specific cell is zero, no current flows through the device. Consequently, the distribution of the output variance is related to the number of activated rows n and can be written as $I \sim N(\sum_r x_r G_r, n\sigma^2)$. To analyze weight dependency, we first assume that all rows are activated in the column, resulting in a constant output variance equal to $256\sigma^2$. However, this prediction relies on a sufficient number of samples, such as those from the entire crossbar array. When considering a single column, the number of samples correlates with the MAC value, since there are not enough samples to support the statistical distribution. For example, if the expected MAC value is 256, which equals the number of rows in the array, the output current becomes deterministic, as all cells must be set to 1. The number of samples is largest when the MAC value is 0 and decreases as the absolute value increases.

For the input test (Fig. S4), the entire array was set to 1 (G_P^+/G_{AP}^-), and a read voltage (0.1 V) was applied to the activated BLs. Due to the variation in the array, even with a fixed number of activated rows, the possible combinations of activated rows could be extensive (C_{256}^n for n activated rows in an array with 256 rows). We randomly selected 500 combinations for each MAC value and repeated the tests on 200 columns.

The weight data dependency was tested by activating all rows in the array and sweeping the weight value from -256 to 256. Similar to the input data test, a single weight value (expected current value) has a vast number of combinations. Due to DtD variation, the same combination for one weight sum value in different columns can yield significantly different output currents. This phenomenon results in varying distributions of real current versus ideal current when tested on one or multiple columns. Consequently, we plotted two different figures to illustrate these distributions: one

obtained from 1e5 combinations for each MAC value in 200 different columns (Fig. S5A) and another from a single column (Fig. S6).

Supplementary Note 6: How Adaptive Quantization Improves the MAC Accuracy

The MAC value of one column in the array is obtained by converting the total output current to voltage using the trans-impedance amplifier (TIA) and then sensed by the output ADC. The quantization process of a non-ideal ADC is denoted by $Quant(I_{input}|\sigma_{ADC})$, in which the average root mean square error (RMSE) measured in Differential Non-Linearity (DNL) of the ADC is 0.112 (LSB). The error of conventional quantization MAC is defined by $Error_{conv} = Quant(I_{real}|\sigma_{ADC}) - Quant(I_{ideal})$. Since the conductance shift sense process allows each cell to be adaptively quantized according to the DSCS-LUT, the expected output value is not directly given by the ideal current. Thus, for the adaptive quantization, the error is defined by $Error_{AQ} = Quant(I_{real}|\sigma_{ADC}) - Quant(I_{AQ})$. We recorded the output MAC error with and without the adaptive quantization scheme in the data dependency tests (Fig. S5B). The errors tend to occur at the sensing threshold of the ADC, with most errors being less than 1 bit (approximately 7.6% error rate). On average, when an error occurs, around 73.66% of the errors can be corrected through adaptive quantization and the RMSE drops 76.79% (Fig. S5C). However, as shown in Supplementary Fig. 5d, due to the limited resolution of the ADC and sensing precision, some data cannot be corrected (12.85%), and in some cases, the error may worsen (13.49%). Overall, the MAC operation accuracy can be significantly increased by applying the adaptive quantization scheme.

Supplementary Note 7: Performance Limitations Imposed by Cycle-to-Cycle Variation

The process of adaptive quantization optimizes the parameters of DNNs by scrutinizing the DSCS-LUT. However, the reliability of the conductance shift sense becomes questionable for devices that exhibit large CtC variations. This is primarily due to the fluctuating conductance observed across different write and read pulses in each cycle. To investigate how CtC variation could constrain the efficacy of adaptive quantization, we artificially augmented the CtC variation of the MTJs in the emulator. The initial conductance of the devices denoted as G_{t_0} is generated with 20% DtD variation in the simulation. A normal distribution between different cycles, $G_{t_n} \sim N(G_{t_0}, \sigma^2)$, was followed by the conductance of each device. The conductance shift sense was conducted in the first write cycle, and subsequent adaptive quantization was performed based on these sensing results.

Fig. S7A illustrates the optimization breakdown of adaptive quantization for the MAC operation. As the CtC variation increases, the proportion of errors that the adaptive quantization can correct reduces. Notably, this percentage drops from 85% under 0% CtC variation to 28% under 100% CtC variation. Fig. S7B presents the accuracy curve of each method tested on the MNIST dataset. At low CtC variation levels, both In-situ training and adaptive quantization can achieve flawless performance. However, as CtC variation increases, the optimization grounded on conductance shift awareness becomes less reliable, leading to a decline in the performance of In-situ training and adaptive quantization. Ultimately, if the CtC variation is exceedingly large, these two methods' optimization becomes entirely dysfunctional. Their performance becomes similar with the VAT method, which relies solely on the device's statistical model and the model's parameter redundancy. As depicted in Fig. S7C, the In-situ training, adaptive quantization, and VAT exhibit similar performance when the CtC variation exceeds 75%.

Adaptive quantization is designed to capture the spatially random yet temporally fixed variation of devices. Hence, it favors devices with low CtC variation. However, our emulation indicates that adaptive quantization can still deliver substantial optimization for devices that suffer from significant CtC variations.

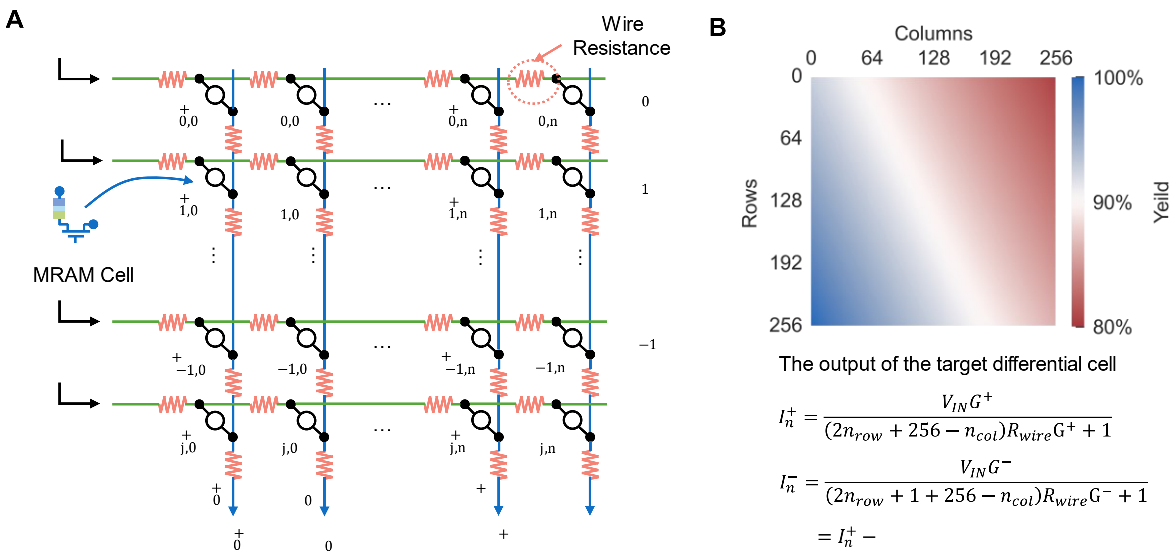

Fig. S1. The IR drop caused by the interconnect wire resistance in the crossbar array. (**A**) the output of the differential cell is obtained by sensing the differential voltage converted from the source line current by the TIA, the conductance of each MTJ in the differential cell is also configured differentially. The wire resistance mainly contributed from the bit line and the source line and is proportional to the wire length. The IR drop can reduce the yield of the differential cell (**B**) the output distorted by the wire resistance can be modeled as a function of the position of the cell in the array and the yield can be plotted.

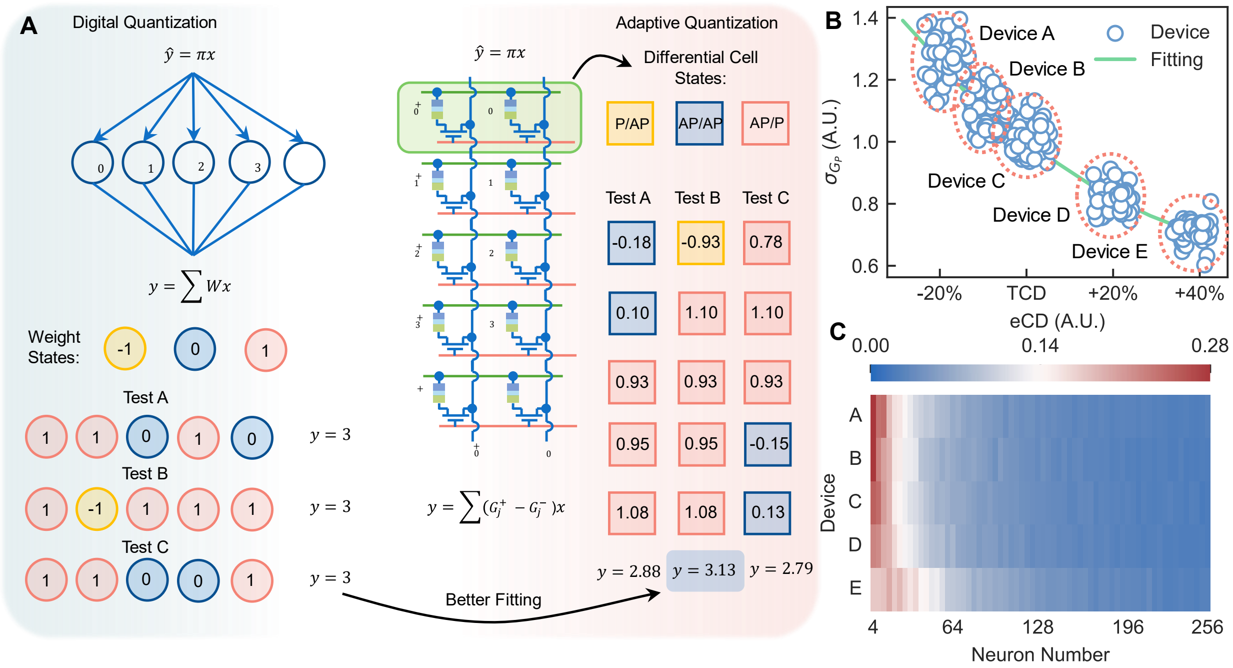

Fig. S2. Linear regression experiment. (**A**) illustration of how digital quantization and adaptive quantization work. In digital quantization, weights are only used as quantized values that are represented by the device states. The quantized value is adjusted for adaptive quantization according to the conductance shift of each particular differential cell. The adaptive quantized model can potentially provide better regression results than conventional digital quantization. (**B**) The conductance variation measured on five batches of MRAM devices fabricated with different eCDs. The fitting curve shows that the scaling variation increases with the decrease of the eCD. (**C**) Linear regression error conducted on five batches of devices. For adaptive quantization, purely increasing the number of neurons/devices can increase the performance of the model, which is not possible for digital quantization models. The result also shows that the larger variation caused by device scaling does not decrease the model performance when an adequate number of neurons is used in the model.

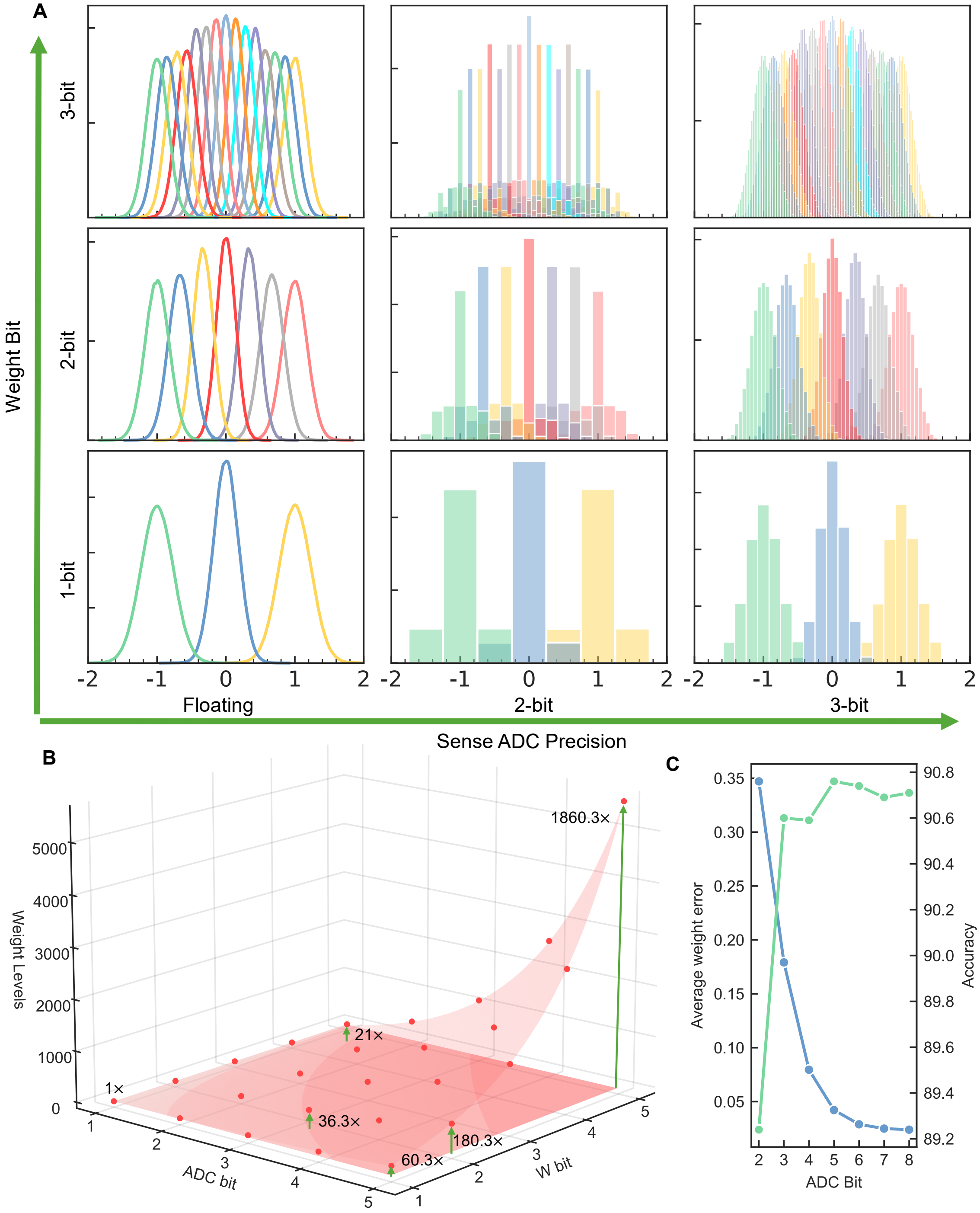

Fig. S3. How weight bit number and conductance shift sensing ADC precision influence the total weight levels. (**A**) Visualization of the weight distribution change, and (**B**) the total weight levels concerning the change of the weight bit and ADC bit precision. (**C**) Increasing the ADC precision can also reduce the average weight mapping error and thus jointly increase the AI inference accuracy. Due to the CtC variation overhead, the effectiveness of increasing the ADC precision is reducing. Thus, the optimal trade-off of the ADC bit is 3.

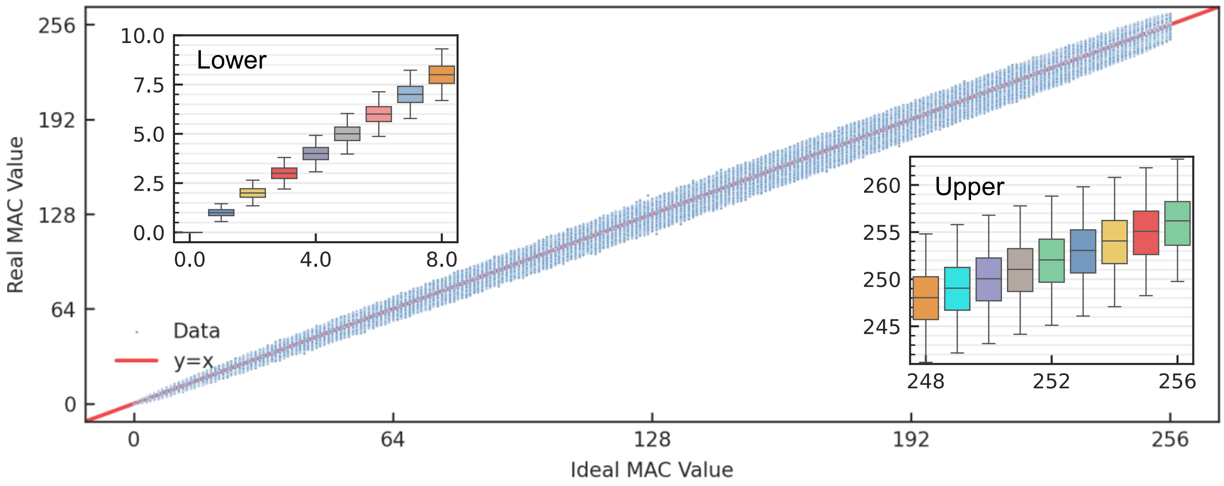

Fig. S4. The MAC error analysis of the input dependency is tested on a 200 column and each MAC value is tested for 1e5 combinations.

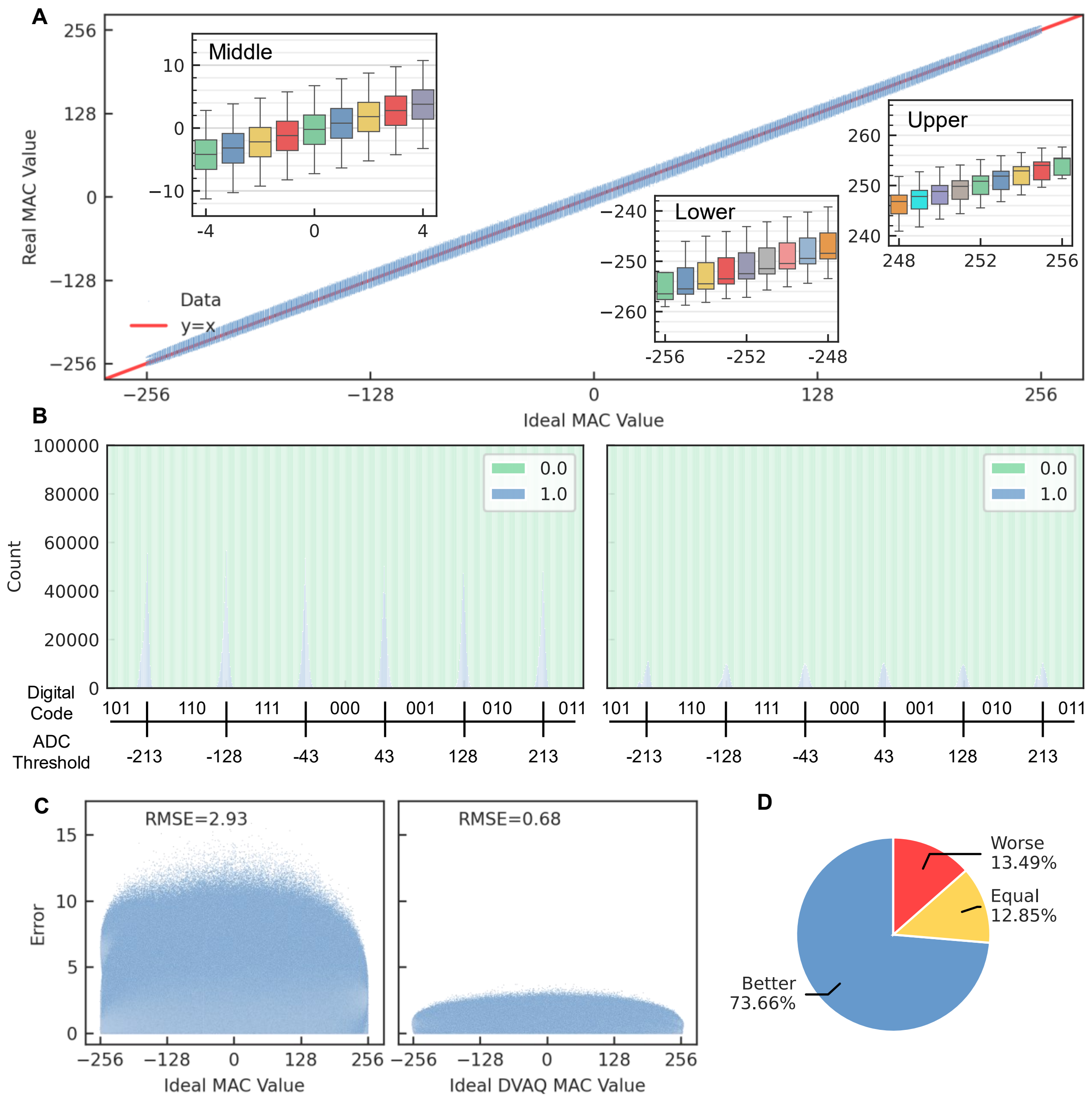

Fig. S5. The MAC error analysis of the weight dependency is tested on 200 columns and each MAC value is tested for 500 combinations. (**A**) The transfer function shows that when tested on multiple columns, the variation is not dependent on the weight value (**B**) the error bit counts in LSB of the digital output code from the ADC before and after the DVAQ calibration, and (**C**) the absolute error before and after the DVAQ. (**D**) When the error occurs, the pie chart shows the percentage of the MAC that is better/equal/worse than the original output.

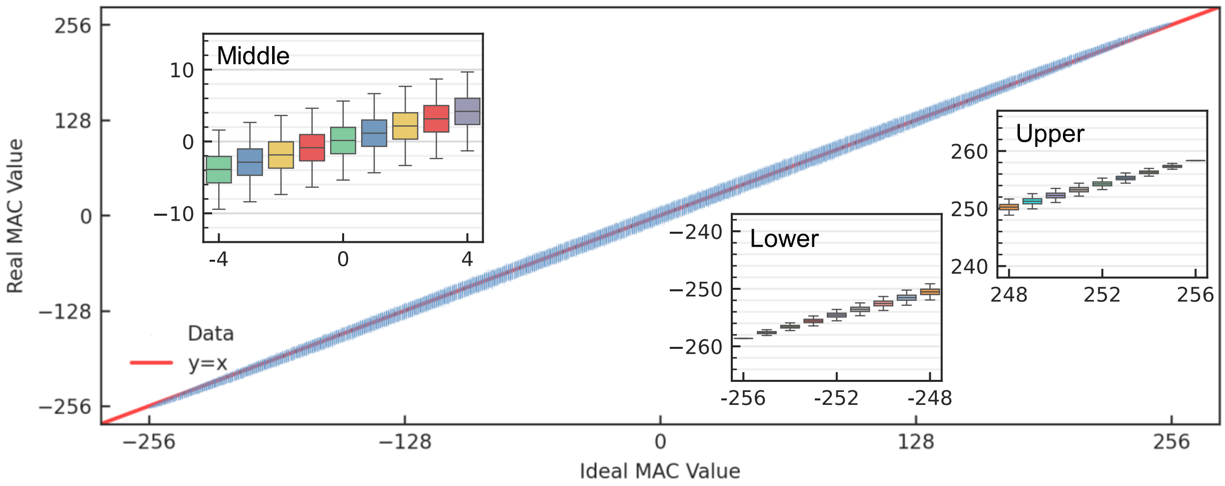

Fig. S6. The MAC error analysis of the weight dependency is tested on a single column and each MAC value is tested for 1e5 combinations.

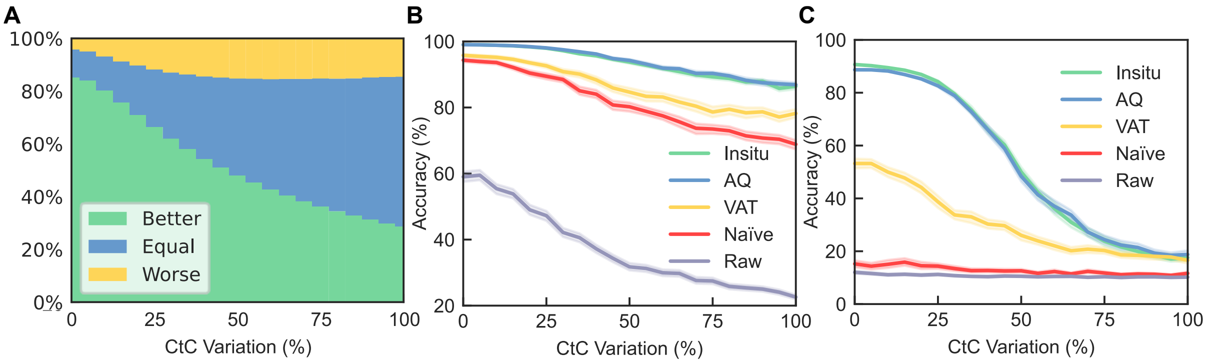

Fig. S7. How can adaptive quantization correct the error when an error occurs in the MAC test. (**A**) Shows the fraction of better, equal, or worse. The CtC variation will reduce the effectiveness of adaptive quantization. When the CtC variation is too large, the fraction of the error that can't be corrected is dominant, making the adaptive quantization unfunctional. (**B**) The accuracy change of each method with different CtC variations in the MNIST dataset using LeNet-5 and (**C**) Cifar-10 using the NIN model.

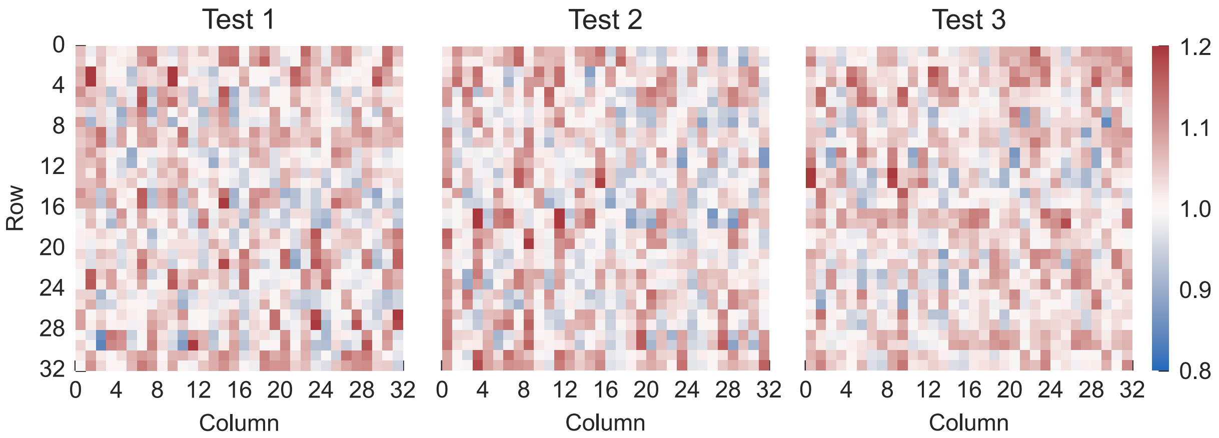

Fig. S8. Resistance bit map of multiple 32 by 32 subregions tested at the low resistance state.

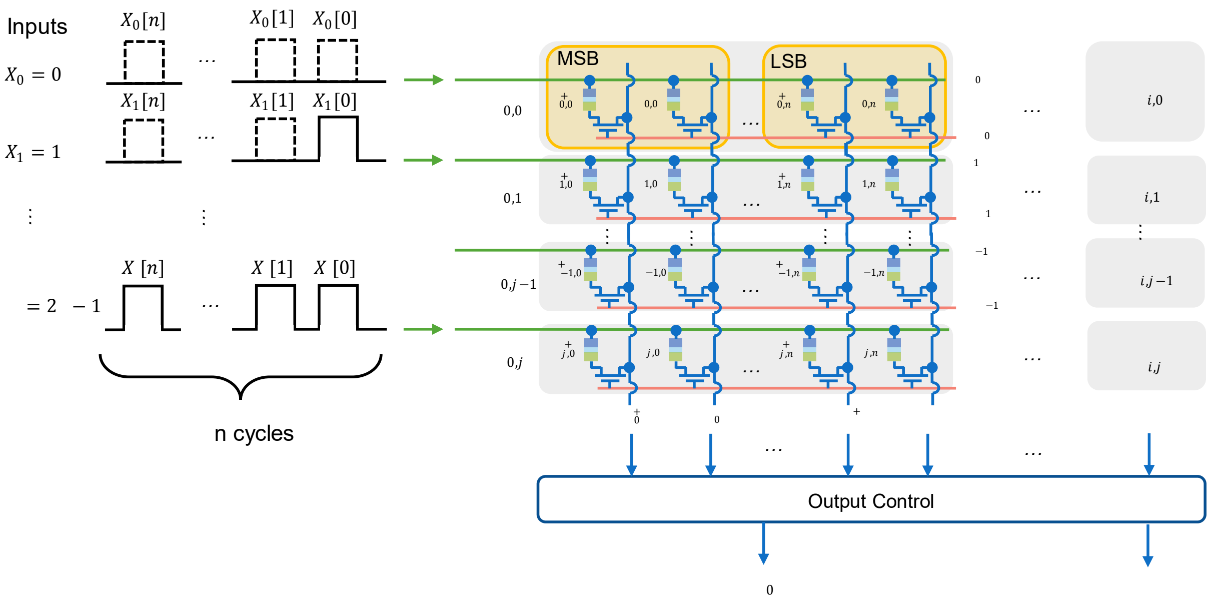

Fig. S9. The MRAM AiMC crossbar array structure and weight mapping illustration.

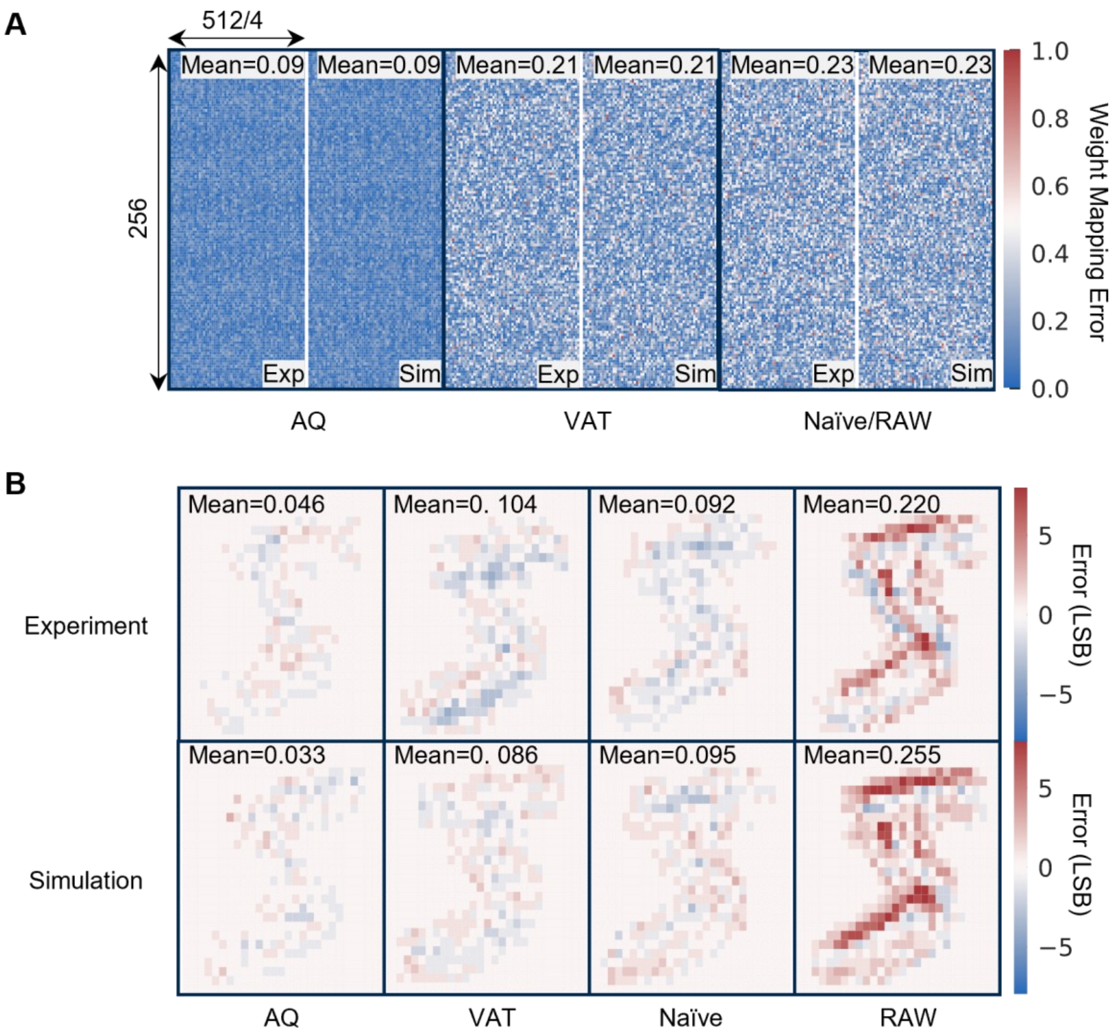

Fig. S10. Comparison between experiment and simulation results on MNIST use TCD devices. (**A**) The weight mapping error of each co-design scheme in experiments and simulations. The mean error values of the simulation are equal to the experiments. (**B**) The output feature map error of the first convolution layer in LeNet. The simulated results have a similar mean error as the experiment.

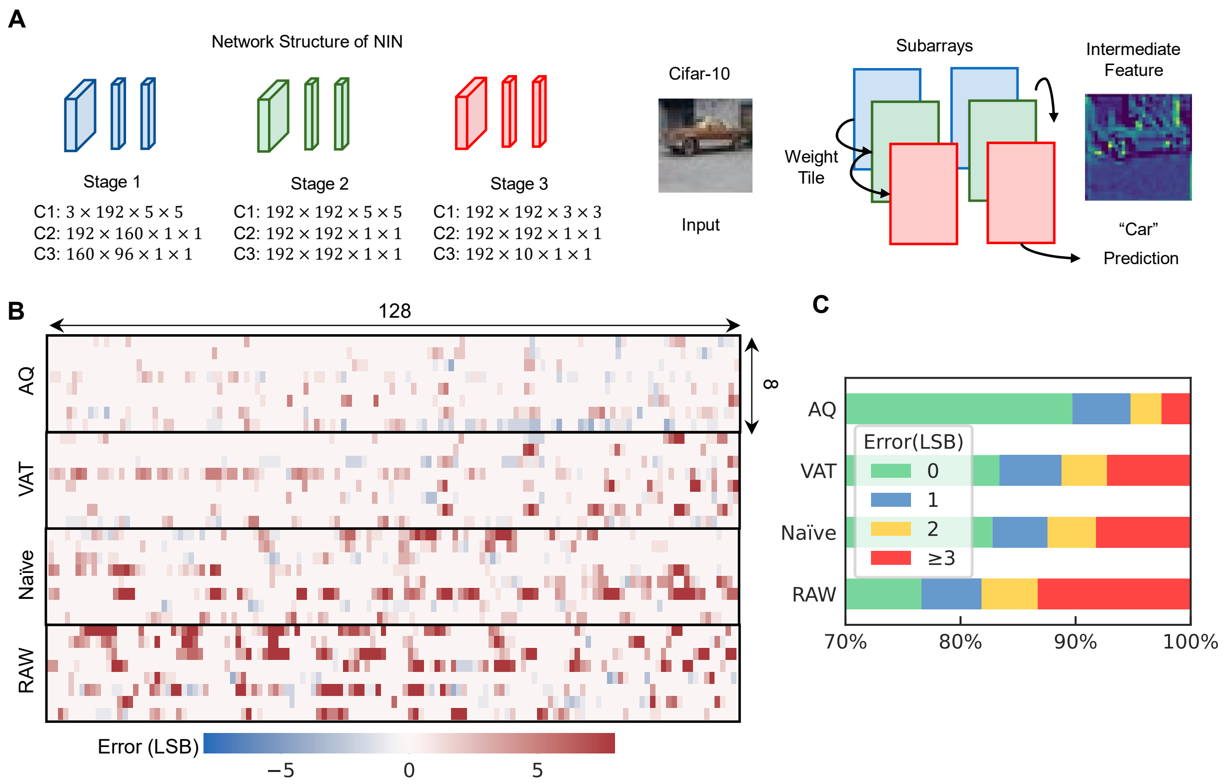

Fig. S11. Weight mapping scheme and error for NIN. (**A**) To map the 2-bit weights in the 256×512 array, the parameters of different kernels and stages are tiled into different subarrays and the partial results will be accumulated in digital values. (**B**) The feature map output error of the last convolutional layer in the model for different schemes and (**C**) the error represented in LSB for different schemes.

Table S1. Variation in different emerging non-volatile memories

	This Work	Nature 22 (*20*)	Science 19 (*5*)	Nature 23 (*6*)	IEDM 22 (*57*)
Technology	MRAM	MRAM	PCM	RRAM	RRAM
Tested device	1 million	8192	~5	65,536	1000
Calibration	No	No	N.A.	Iterative	N.A.
Cycle-to-cycle variation	0.56%	N.A.	0.4%	0.12%	3%
Device-to-device variation	3.28%	12.3%	N.A.*	0.04%†	9.4%

* Not enough number of tested devices to conclude

† Devices are programmed to target conductance and then measure

Table S2. Accuracy changes of different co-design schemes

	This Work	Nature 22 (*20*)	Science 23 (*58*)	JSSC 22 (*59*)	Nat Electron 22 (*60*)
Technology	MRAM	MRAM	RRAM	RRAM	RRAM
Array size	256x512	64x64	256x512	256x256	8x8x32
ADC bits	3-bit	4-bit	8-bit	4-bit	6-bit
NN demo	MNIST				
Calibration	Off-chip	Off-chip	On-chip*	On-chip*	On-chip*
Accuracy	99.36%	95.24%	95.8%	95.8%	97.04%
Acc. drop compared with the ideal case	-0.05%	2.01%	0.8%	2.7%	0.81%

*write-verify scheme

Table S3. **Example of a DSCS-LUT for a single cell**

State	Device Positive (+)	Device Negative (-)	Output Conductance			Sensed shift	Calibrated conductance	Calibrated Error(abs.)
			On chip	Standard	Error (abs.)			
1	$G_P^+ = 1.599$*	$G_{AP}^- = 1.006$	0.593	0.8	0.207	-0.26	0.54	0.053
0	$G_{AP}^+ = 0.881$	$G_{AP}^- = 1.006$	-0.125	0	0.125	-0.13	-0.13	0.005
-1	$G_{AP}^+ = 0.881$	$G_P^- = 1$	-0.916	-0	0.116	-0.13	-0.93	0.014

*The conductance is normalized.

**In this case, the conductance of each device is measured by external circuits and the sensed shift is down sampled to 3-bit. For actual implementation, the sensing is done by on-chip circuits and this table will only contain the state and calibrated conductance.

REFERENCES AND NOTES

1. A. Sebastian, M. Le Gallo, R. Khaddam-Aljameh, E. Eleftheriou, Memory devices and applications for in-memory computing. *Nat. Nanotechnol.* **15**, 529–544 (2020).

2. Y. Lecun, 1.1 Deep Learning Hardware: Past, Present, and Future," in *2019 IEEE International Solid-State Circuits Conference*, (ISSCC, San Francisco, CA, USA, 2019).

3. N. Verma, H. Jia, H. Valavi, Y. Tang, M. Ozatay, L. Y. Chen, B. Zhang, P. Deaville, In-memory computing: Advances and prospects. *IEEE J. Solid-State Circuits* **11**, 43–55 (2019).

4. M. Rizzi, N. Ciocchini, A. Montefiori, M. Ferro, P. Fantini, A. L. Lacaita, D. Ielmini, Cell-to-cell and cycle-to-cycle retention statistics in phase-change memory arrays. *IEEE Trans. Electron Devices* **62**, 2205–2211 (2015).

5. K. Ding, J. Wang, Y. Zhou, H. Tian, L. L. Lu, R. Mazzarello, C. Jia, W. Zhang, F. Rao, E. Ma, Phase-change heterostructure enables ultralow noise and drift for memory operation. *Science* **366**, 210–215 (2019).

6. M. Rao, H. Tang, J. Wu, W. Song, M. Zhang, W. Yin, Y. Zhuo, F. Kiani, B. Chen, X. Jiang, H. Liu, H.-Y. Chen, R. Midya, F. Ye, H. Jiang, Z. Wang, M. Wu, M. Hu, H. Wang, Q. Xia, N. Ge, J. Li, J. J. Yang, Thousands of conductance levels in memristors integrated on CMOS. *Nature* **615**, 823–829 (2023).

7. M. Zhao, H. Wu, B. Gao, X. Sun, Y. Liu, P. Yao, Y. Xi, X. Li, Q. Zhang, K. Wang, S. Yu, H. Qian, Characterizing endurance degradation of incremental switching in analog RRAM for neuromorphic systems. *2018 IEEE International Electron Devices Meeting,* (*IEDM*, San Francisco, CA, USA, 2019).

8. E. Esmanhotto, L. Brunet, N. Castellani, D. Bonnet, T. Dalgaty, High-density 3D monolithically integrated multiple 1T1R multi-level-cell for neural networks, *2020 IEEE International Electron Devices Meeting* (*IEDM*, San Francisco, CA, 2020).

9. C. Nail, G. Molas, P. Blaise, G. Piccolboni, B. Sklenard, C. Cagli, M. Bernard, A. Roule, M. Azzaz, E. Vianello, C. Carabasse, R. Berthier, D. Cooper, C. Pelissier, T. Magis, G. Ghibaudo, C. Vallee, D. Bedeau, O. Mosendz, B. De Salvo, L. Perniola, Understanding RRAM endurance, retention and window margin trade-off using experimental results and simulations. *2016 IEEE International Electron Devices Meeting* (IEDM, San Francisco, CA, 2017).

10. A. Chanthbouala, V. Garcia, R. O. Cherifi, K. Bouzehouane, S. Fusil, X. Moya, S. Xavier, H. Yamada, C. Deranlot, N. D. Mathur, M. Bibes, A. Barthélémy, J. Grollier, A ferroelectric memristor. *Nat. Mater.* **11**, 860–864 (2012).

11. J. Ma, J. Ma, Q. Zhang, R. Peng, J. Wang, C. Liu, M. Wang, N. Li, M. Chen, X. Cheng, P. Gao, L. Gu, L. Q. Chen, P. Yu, J. Zhang, C. W. Nan, Controllable conductive readout in self-assembled, topologically confined ferroelectric domain walls. *Nat. Nanotechnol.* **13**, 947–952 (2018).

12. Y. J. Song, J. H. Lee, S. H. Han, H. C. Shin, K. H. Lee, K. Suh, D. E. Jeong, G. H. Koh, S. C. Oh, J. H. Park, S. O. Park, B. J. Bae, O. I. Kwon, K. H. Hwang, B. Y. Seo, Y. K. Lee, S. H. Hwang, D. S. Lee, Y. Ji, K. C. Park, G. T. Jeong, H. S. Hong, K. P. Lee, H. K. Kang, E. S. Jung, Demonstration of highly manufacturable STT-MRAM embedded in 28nm logic. *2018 IEEE International Electron Devices Meeting,* (IEDM, San Francisco, CA, 2019).

13. V. B. Naik, K. Yamane, T. Y. Lee, J. Kwon, R. Chao, J. H. Lim, N. L. Chung, B. Behin-Aein, L. Y. Hau, D. Zeng, Y. Otani, C. Chiang, Y. Huang, L. Pu, S. H. Jang, W. P. Neo, H. Dixit, S. K. L. C. Goh, E. H. Toh, T. Ling, J. Hwang, J. W. Ting, R. Low, L. Zhang, C. G. Lee, N. Balasankaran, F. Tan, K. W. Gan, H. Yoon, G. Congedo, J. Mueller, B. Pfefferling, O. Kallensee, A. Vogel, V. Kriegerstein, T. Merbeth, C. S. Seet, S. Ong, J. Xu, J. Wong, Y. S. You, S. T. Woo, T. H. Chan, E. Quek, S. Y. Siah, JEDEC-qualified highly reliable 22nm FD-SOI embedded MRAM for low-power industrial-grade, and extended performance towards automotive-grade-1 applications. *2020 IEEE International Electron Devices Meeting,* (IEDM, San Francisco, CA, 2020).

14. Y. C. Shih, C. F. Lee, Y. A. Chang, P. H. Lee, H. J. Lin, Y. L. Chen, C. P. Lo, K. F. Lin, T. W. Chiang, Y. J. Lee, K. H. Shen, R. Wang, W. Wang, H. Chuang, E. Wang, Y. Der Chih, J. Chang,

"A reflow-capable, embedded 8Mb STT-MRAM macro with 9nS read access time in 16nm FinFET logic CMOS process" in *2020 IEEE International Electron Devices Meeting,* (IEDM, San Francisco, CA, 2020).

15. D. Edelstein, M. Rizzolo, D. Sil, A. Dutta, J. Debrosse, M. Wordeman, A. Arceo, I. C. Chu, J. Demarest, E. R. J. Edwards, E. R. Evarts, J. Fullam, A. Gasasira, G. Hu, M. Iwatake, R. Johnson, V. Katragadda, T. Levin, J. Li, Y. Liu, C. Long, T. Maffitt, S. Mcdermott, S. Mehta, V. Mehta, D. Metzler, J. Morillo, Y. Nakamura, S. Nguyen, P. Nieves, V. Pai, R. Patlolla, R. Pujari, R. Southwick, T. Standaert, O. Van Der Straten, H. Wu, C.-C. Yang, D. Houssameddine, J. M. Slaughter, D. C. Worledge, A 14 Nm embedded STT-MRAM CMOS technology. *2020 IEEE International Electron Devices Meeting,* (IEDM, San Francisco, CA, 2020).

16. W. Wan, R. Kubendran, C. Schaefer, S. B. Eryilmaz, W. Zhang, D. Wu, S. Deiss, P. Raina, H. Qian, B. Gao, S. Joshi, H. Wu, H. S. P. Wong, G. Cauwenberghs, A compute-in-memory chip based on resistive random-access memory. *Nature* **608**, 504–512 (2022).

17. B. Zhang, P. Deaville, N. Verma, Statistical computing framework and demonstration for in-memory computing systems. *DAC '22: 59th ACM/IEEE Design Automation Conference*, (IEDM, San Francisco, CA, 2022).

18. K. Garello, B. Verhoef, R. Degraeve, S. Van Beek, D. Crotti, F. Yasin, S. Couet, G. Jayakumar, A Papistas, P. Debacker, R. Lauwereins, W. Dehaene, G.S. Kar, S. Cosemans, A. Mallik, D. Verkest J. Doevenspeck, SOT-MRAM based analog in-memory computing for DNN inference, in *2020 IEEE Symposium on VLSI Technology*, (IEEE, Honolulu, HI, USA, 2020).

19. J. M. Goodwill, N. Prasad, B. D. Hoskins, M. W. Daniels, A. Madhavan, L. Wan, T. S. Santos, M. Tran, J. A. Katine, P. M. Braganca, M. D. Stiles, J. J. McClelland, Implementation of a binary neural network on a passive array of magnetic tunnel junctions. *2022 IEEE 33rd Magnetic Recording Conference*, (IEEE, Milpitas, CA, 2022).

20. S. Jung, H. Lee, S. Myung, H. Kim, S. Keun Yoon, S.-W. Kwon, Y. Ju, M. Kim, W. Yi, S. Han, B. Kwon, B. Seo, K. Lee, G.-H. Koh, K. Lee, Y. Song, C. Choi, D. Ham, S. Joon Kim, J. A.

Paulson, A crossbar array of magnetoresistive memory devices for in-memory computing. *Nature* **601**, 211–216 (2022).

21. C.-C. Chang, S.-T. Li, T.-L. Pan, C.-M. Tsai, I.-T. Wang, T.-S. Chang, T.-H. Hou, Device quantization policy in variation-aware in-memory computing design. *Sci. Rep.* **12**, 112 (2022).

22. P. Yao, H. Wu, J. Tang, Q. Zhang, W. Zhang, J. J. Yang, H. Qian, Fully hardware-implemented memristor convolutional neural network. *Nature* **577**, 641–646 (2020).

23. S. Yu, W. Shim, X. Peng, Y. Luo, RRAM for compute-in-memory: From inference to training. *IEEE Trans. Circuits Syst. I: Regul. Pap.* **68**, 2753–2765 (2021).

24. J. Doevenspeck, P. Vrancx, N. Laubeuf, A. Mallik, P. Debacker, D. Verkest, R. Lauwereins, W. Dehaene, "Noise tolerant ternary weight deep neural networks for analog in-memory inference" in *Proceedings of the International Joint Conference on Neural Networks* (Institute of Electrical and Electronics Engineers Inc., Shenzhen, China, 2021).

25. B. Zhang, L. Y. Chen, N. Verma, Neural network training with stochastic hardware models and software abstractions. *IEEE Trans. Circuits Syst. I: Regul. Pap.* **68**, 1532–1542 (2021).

26. H. Cai, Y. Guo, B. Liu, M. Zhou, J. Chen, X. Liu, J. Yang, Proposal of analog in-memory computing with magnified tunnel magnetoresistance ratio and universal STT-MRAM Cell. *IEEE Trans. Circuits Syst. I: Regul. Pap.* **69**, 1519–1531 (2022).

27. Z.-J. Bian, Y.-N. Guo, B. Liu, H. Cai, "In-MRAM computing elements with single-step convolution and fully connected for BNN/TNN" in *2021 IEEE International Conference on Integrated Circuits, Technologies and Applications* (ICTA, Zhuhai, China, 2021).

28. P. Deaville, B. Zhang, N. Verma, "A 22nm 128-Kb MRAM row/column-parallel in-memory computing macro with memory-resistance boosting and multi-column ADC readout" in *2022 IEEE Symposium on VLSI Technology and Circuits*, (VLSI Technology and Circuits, Honolulu, HI, 2022).

29. J. Kaiser, W. A. Borders, K. Y. Camsari, S. Fukami, H. Ohno, S. Datta, Hardware-aware in situ learning based on stochastic magnetic tunnel junctions. *Phys. Rev. Appl.* **17**, 014016 (2022).

30. Z. Xiao, V. B. Naik, S. K. Cheung, J. H. Lim, J.-H. Kwon, Z. Ren, Z. Wang, Q. Shao, “Device variation-aware adaptive quantization for MRAM-based accurate in-memory computing without on-chip training” in *Technical Digest - International Electron Devices Meeting,* (IEDM, San Francisco, CA, 2022).

31. Y. LeCun, L. Bottou, Y. Bengio, P. Haffner, Gradient-based learning applied to document recognition. *Proc. IEEE* **86**, 2278–2323 (1998).

32. M. Lin, Q. Chen, S. Yan, “Network in network” in *2nd International Conference on Learning Representations*, (ICLR, Banff, AB, 2013).

33. A. Krizhevsky, Learning multiple layers of features from tiny images. University of Toronto, Toronto. (2009).

34. W. Song, M. Rao, Y. Li, C. Li, Y. Zhuo, F. Cai, M. Wu, W. Yin, Z. Li, Q. Wei, S. Lee, H. Zhu, L. Gong, M. Barnell, Q. Wu, P. A. Beerel, M. S.-W. Chen, N. Ge, M. Hu, Q. Xia, J. J. Yang, Programming memristor arrays with arbitrarily high precision for analog computing. *Science* **383**, 903–910 (2024).

35. H. Cai, Z. Bian, Y. Hou, Y. Zhou, J.-L. Cui, Y. Guo, X. Tian, B. Liu, X. Si, Z. Wang, J. Yang, W. Shan, “A 28nm 2Mb STT-MRAM computing-in-memory macro with refined bit-cell and 22.4~41.5 TOPS/W for AI inference” in *IEEE International Solid-State Circuits Conference*, (ISSCC, San Francisco, CA, USA, 2023).

36. Y.-C. Chiu, “A 22nm 4Mb STT-MRAM data-encrypted near-memory computation macro with a 192GB/S read-and-decryption bandwidth and 25.1–55.1TOPS/W 8b MAC for AI operations” in *2022 IEEE International Solid-State Circuits Conference* (ISSCC, San Francisco, CA, USA, 2022).

37. P. Deaville, B. Zhang, L.-Y. Chen, N. Verma, “A maximally row-parallel MRAM in-memory-computing macro addressing readout circuit sensitivity and area” in *ESSCIRC 2021 - IEEE 47th European Solid State Circuits Conference* (ESSCIRC, Grenoble, France, 2021).

38. P. Y. Chen, X. Peng, S. Yu, NeuroSim: A circuit-level macro model for benchmarking neuro-inspired architectures in online learning. *IEEE Trans. Comput.-Aided Des. Integr. Circuits Syst.* **37**, 3067–3080 (2018).

39. S. Zhou, Y. Wu, Z. Ni, X. Zhou, H. Wen, Y. Zou, DoReFa-Net: Training low bitwidth convolutional neural networks with low bitwidth gradients. *arXiv* **1606**, 6160 (2016).

40. Y. Bengio, N. Léonard, A. Courville, Estimating or propagating gradients through stochastic neurons for conditional computation. *arXiv* **1308**, 3432 (2013).

41. I. Loshchilov, F. Hutter, “Decoupled weight decay regularization” in *7th International Conference on Learning Representations,* (ICLR, New Orleans, 2019).

42. Z. He, J. Lin, R. Ewetz, J. S. Yuan, D. Fan, “Noise injection adaption: End-to-End ReRAM crossbar non-ideal effect adaption for neural network mapping” in *Proceedings - Design Automation Conference* (IEEE, New Orleans, 2019).

43. S. Lee, J. Jeon, K. Eom, C. Jeong, Y. Yang, J. Y. Park, C. B. Eom, H. Lee, Variance-aware weight quantization of multi-level resistive switching devices based on Pt/LaAlO3/SrTiO3 heterostructures. *Sci. Rep.* **12**, 1–10 (2022).

44. L. X. Han, Y. C. Xiang, P. Huang, G. H. Yu, R. Z. Han, X. Y. Liu, J. F. Kang, “Novel weight mapping method for reliable NVM based neural network” in *IEEE International Reliability Physics Symposium Proceedings* (IEEE, Monterey, CA, 2021).

45. Y. Luo, X. Peng, R. Hatcher, T. Rakshit. A variation robust inference engine based on STT-MRAM with parallel read-out" in *2020 IEEE International Symposium on Circuits and Systems* (ISCAS, Seville, Spain, 2020).

46. Y. Geng, B. Gao, Q. Zhang, W. Zhang, P. Yao, Y. Xi, Y. Lin, J. Chen, J. Tang, H. Wu, H. Qian, "An on-chip layer-wise training method for RRAM based computing-in-memory chips" in *2021 Design, Automation & Test in Europe Conference & Exhibition* (DATE, Grenoble, France, 2021).

47. G. Hinton, O. Vinyals, J. Dean, Distilling the knowledge in a neural network. *arXiv*.1503.02531 (2015).

48. G. Hinton, G. Brain, The forward-forward algorithm: Some preliminary investigations. *arXiv.* 2212.13345 (2022).

49. S. Okumura, M. Yabuuchi, K. Hijioka, K. Nose, "A ternary based bit scalable, 8.80 TOPS/W CNN accelerator with many-core processing-in-memory architecture with 896K synapses/mm2" in *2019 Symposium on VLSI Circuits,* (IEEE, Kyoto, Japan, 2019).

50. W. Alexander, The ternary computer. *Electron. Power* **10**, 36 (1964).

51. Y.-C. Chiu, W.-S. Khwa, C.-S. Yang, S.-H. Teng, H.-Y. Huang, F.-C. Chang, Y. Wu, Y.-A. Chien, F.-L. Hsieh, C.-Y. Li, G.-Y. Lin, P.-J. Chen, T.-H. Pan, C.-C. Lo, R.-S. Liu, C.-C. Hsieh, K.-T. Tang, M.-S. Ho, C.-P. Lo, Y.-D. Chih, T.-Y. J. Chang, M.-F. Chang, A CMOS-integrated spintronic compute-in-memory macro for secure AI edge devices. *Nat. Electron.* **2023**, 1–10 (2023).

52. Z. Jing, B. Yan, Y. Yang, R. Huang, VSDCA: A voltage sensing differential column architecture based on 1T2R RRAM array for computing-in-memory accelerators. *IEEE Trans. Circuits Syst. I: Regul. Pap.* **69**, 1–14 (2022).

53. Z. Liu, K.-T. Cheng, D. Huang, E. Xing, Z. Shen, Nonuniform-to-uniform quantization: Towards accurate quantization via generalized straight-through estimation. *arXiv*.2111.14826 (2021).

54. S. K. Esser, J. L. McKinstry, D. Bablani, R. Appuswamy, D. S. Modha, Learned step size quantization. IBM Research, San Jose, California, (2019).

55. B. Jacob, S. Kligys, B. Chen, M. Zhu, M. Tang, A. Howard, H. Adam, D. Kalenichenko, Quantization and training of neural networks for efficient integer-arithmetic-only inference. (2017).

56. M. Geiger, L. Petrini, M. Wyart, Landscape and training regimes in deep learning. *Phys. Rep.* **924**, 1–18 (2021).

57. X. Li, B. Gao, B. Lin, R. Yu, H. Zhao, Z. Wang, Q. Qin, J. Tang, Q. Zhang, X. Li, Z. Hao, X. Li, D. Kong, L. Ma, N. Deng, H. Qian, H. Wu, \First demonstration of homomorphic encryption using multi-functional RRAM arrays with a novel noise-modulation scheme in *2022 International Electron Devices Meeting* (IEDM, San Francisco, CA*,* 2022).

58. W. Zhang, P. Yao, B. Gao, Q. Liu, D. Wu, Q. Zhang, Y. Li, Q. Qin, J. Li, Z. Zhu, Y. Cai, D. Wu, J. Tang, H. Qian, Y. Wang, H. Wu, Edge learning using a fully integrated neuro-inspired memristor chip. *Science* **381**, 1205–1211 (2023).

59. J.-H. Yoon, M. Chang, W.-S. Khwa, Y.-D. Chih, M.-F. Chang, A. Raychowdhury, A 40-nm, 64-Kb, 56.67 TOPS/W voltage-sensing computing-in-memory/digital RRAM macro supporting iterative write with verification and online read-disturb detection. *IEEE J. Solid-State Circuits* **57** 1 (2022).

60. Q. Huo, Y. Yang, Y. Wang, D. Lei, X. Fu, Q. Ren, X. Xu, Q. Luo, G. Xing, C. Chen, X. Si, H. Wu, Y. Yuan, Q. Li, X. Li, X. Wang, M. F. Chang, F. Zhang, M. Liu, A computing-in-memory macro based on three-dimensional resistive random-access memory. *Nat. Electron.* **5**, 469–477 (2022).
